# Spring peaks and autumn troughs identified in peripheral inflammatory markers during the peripartum period

**DOI:** 10.1038/s41598-019-51527-9

**Published:** 2019-10-25

**Authors:** Hanna E. Henriksson, Richard A. White, Stavros I. Iliadis, Emma Fransson, Fotios C. Papadopoulos, Inger Sundström-Poromaa, Alkistis Skalkidou

**Affiliations:** 1 Department of Women’s and Children’s Health, Uppsala University, Uppsala, Sweden; 2 Norwegian Institute of Public Health, Oslo, Norway; 3 Department of Microbiology, Tumor and Cell Biology, Karolinska Institutet, Stockholm, Sweden; 4 Department of Neuroscience, Psychiatry, Uppsala University, Uppsala, Sweden

**Keywords:** Assay systems, Chemokines

## Abstract

Seasonal variations have recently been described in biomarkers, cell types, and gene expression associated with the immune system, but so far no studies have been conducted among women in the peripartum period. It is of note that pregnancy complications and outcomes, as well as autoimmune diseases, have also been reported to exhibit seasonal fluctuations. We report here a clear-cut seasonal pattern of 23 inflammatory markers, analysed using proximity-extension assay technology, in pregnant women. The inflammatory markers generally peaked in the spring and had a trough in the autumn. During the postpartum period we found seasonality in one inflammatory marker, namely monocyte chemotactic protein 4 (MCP-4). Our findings suggest that seasonal variations in peripheral inflammatory markers are only observed during pregnancy. The results of this study could be valuable to professionals working within the field of immunology-related areas, and provide insight for the understanding of obstetric complications.

## Introduction

The interest in how the change of seasons affects disease and well-being dates back to ancient Greece^1^. In the present time, seasonal variations are suggested in pregnancy complications and in outcomes such as preterm birth and preeclampsia^2^, conditions that have also been associated with altered immunity^3,4^. Spontaneous preterm birth has been reported to occur more often during summer months^5^, but no seasonality has been observed among induced preterm births. Some studies report a second peak of preterm births during winter^6^, while gestational diabetes and gestational hypertension are more common during the warm months of spring and summer^2,7,8^. Although current data are contradictory, women giving birth in the last three months of the year have been reported to be more likely to develop postpartum depressive symptoms^9,10^. Autoimmune disease activity is influenced by seasonally changing environmental factors and several conditions with immunological and inflammatory components in their aetiology, including multiple sclerosis, systemic lupus erythematosus, psoriasis, and rheumatoid arthritis, display seasonal patterns^11^.

From an immunological perspective, pregnancy is a rather distinct condition as semi-allogeneic tissues are being developed in the woman’s body without stimulating a detrimental immune response against the foetus, while still maintaining a barrier against pathogens. Several mechanisms allowing the immunologically and genetically foreign foetus to survive to term have been suggested^12^, and a key role of maternal regulatory T lymphocytes (T_reg_) in suppressing immune response against the foetus has been described^13^. Furthermore, during pregnancy, there are three immunological phases which are characterised based on the macrophage milieu^14^. Macrophages are monocyte-derived plastic cells, which orchestrate the immune response^15^ and can shift from an M1 state with antigen-presenting capacity and a T cell response skewed toward the more pro-inflammatory T helper type 1 (Th1), to an M2 state associated with immunosuppressive qualities and T helper type 2 (Th2) immune response^16,17^. Early pregnancy has been suggested to be dominated by an M1 phase, as pro-inflammatory cytokines play an important role in the implantation and placentation^16,18^. In the second trimester, as the placenta is fully developed, an anti-inflammatory M2 phase follows, allowing rapid foetal growth and which may counteract preterm contractions^16^. This phase continues into the third trimester, but then studies have reported a last pro-inflammatory M1 phase just prior to parturition, suggested to aid in cervix ripening, uterine contractions, and placenta expulsion^19–21^. During the postpartum period, a rapid reversal of the pregnancy-associated immunological alterations occurs. Specifically, studies report a shift towards the Th1 direction and a reversal in the cytokine pattern in the first weeks following childbirth^22,23^, often resulting in the onset or exacerbation of various autoimmune diseases in the postpartum period^23^. The regulatory mechanisms of these adaptive changes remain partly unknown. The implication of sex steroid hormones such as human chorionic gonadotropin, oestriol, eostradiol, and progesterone, which modulate the number of T_reg_ cells has been suggested^24,25^.

Preterm birth has been associated with elevated levels of pro-inflammatory cytokines, such as interleukin (IL)-6, IL-1β and tumor necrosis factor (TNF)-α^26^, which is supported by results indicating an M1-like polarisation of the decidua during spontaneous preterm birth^27^. Similarly, there is evidence of augmented inflammation in the pathophysiology of preeclampsia, involving TNF-α and interferon (IFN)-γ^28^. In women with gestational diabetes, inflammatory markers such as IL-6, IL-10, C-reactive protein, and TNF-α have been reported elevated both in the third trimester and six months postpartum^29^. Interestingly, although major depressive disorder in the general population has been associated with elevated levels of pro-inflammatory cytokines^30^, evidence is contradictory regarding peripartum depressive symptoms with both higher and lower levels of inflammatory markers reported in pregnancy^31–33^. Significant differences in cytokine levels between pregnant Hispanic and African American women, also points to the importance of considering ethnicity and setting when planning studies^33^.

Seasonal variations have recently been described in biomarkers, cell types, and gene expression associated with the immune system^34–38^. A seasonal expression of more than 4000 protein-coding mRNAs in white blood cells and adipose tissue has been reported, with the winter dominated by a pro-inflammatory transcriptomic profile^34^. Interestingly, the seasonal pattern was inverted between the Northern and Southern hemispheres^34^. Liu and Taioli^35^ reported an increased pro-inflammatory profile in winter–spring, compared with summer–autumn, with elevated levels of neutrophils, C-reactive protein, and white blood cells. The production of TNF-α, IL-1β, and IL-6 has been reported to peak in summer^38^. In a European sample, the analysis of full blood count data revealed seasonal patterns in lymphocytes, monocytes, neutrophils, and erythrocytes^34^. Lymphocytes had a pattern with a trough in the spring and a peak in the autumn, while the latter three cell types followed a reversed pattern.

Considering the significant alterations characterising the maternal immune system during pregnancy and the postpartum period, and taking into account the previously described immunological and clinical impact of seasons, it would be of interest to specifically investigate possible fluctuation in the levels of inflammatory markers during gestation and after childbirth. We hypothesise that, during pregnancy, any potential seasonal alterations in inflammatory markers may be attenuated by the vast alterations in the immune system and the large interpersonal variation. On the contrary, in the postpartum period, a seasonal pattern may be present, as the immune system rapidly normalises to a non-pregnant state. To date, no such studies exist in the literature.

Thus, the aim of this study was to investigate the occurrence of seasonal variation in peripheral levels of inflammatory markers during late pregnancy and the early postpartum period.

## Methods

### Subjects

The current study was undertaken as part of the BASIC project, an on-going population-based study at Uppsala University Hospital, Sweden. The primary aim of the study is to investigate correlates of affective symptoms during pregnancy and after childbirth. Women who register for the routine ultrasound examination at the hospital around gestational week 17 were asked to participate. Exclusion criteria were age less than 18 years, not being able to adequately communicate in Swedish, protected identity, blood borne infectious diseases, and non-viable pregnancies. The BASIC study mainly collects data through web surveys sent out at the time of consent (around gestational week 17), at gestational weeks 32, as well as at 6 weeks, 6 months, and 12 months postpartum. The surveys contain, *inter alia*, the Edinburgh Postnatal Depression Scale (EPDS)^39^. The EPDS is a validated, self-administered questionnaire aimed to study depressive symptoms during pregnancy and the postpartum^40,41^. The medical records were used to retrieve information such as date of childbirth, newborn sex, and data on several obstetric variables.

The current cross-sectional study included participants who were invited to the research laboratory of the Department of Obstetrics and Gynaecology at approximately gestational week 38 and 8 weeks postpartum. The overall aim of this sub-study was to more thoroughly assess a selection of the participants, who were invited based on their EPDS score at gestational week 32 and/or 6 weeks postpartum (participation at both time-points was not mandatory). Participants scoring ≥12 and ≤6, and those on selective serotonin reuptake inhibitors (SSRI) medication were targeted. Two-hundred-twenty-one pregnant and 192 postpartum women participated. A non-fasting blood sample was collected, the Mini International Neuropsychiatric Interview (M.I.N.I.) was conducted, and the participants filled out the EPDS.

Adding to the pregnancy samples collected at the research laboratory, participants who underwent an elective caesarean section (n = 117) during the years 2010–2014 were also specifically asked for participation in order to increase the number of fasting pregnancy samples. These participants were asked to fill out the EPDS and provide a fasting blood sample prior to the surgical procedure. This resulted in a total of 338 (221 + 117) blood samples collected from pregnant women.

### Ethical considerations

The study protocol has been approved by the Regional Ethical Review Board in Uppsala, Sweden (Dnr 2009/171) and the study was conducted in accordance with the Declaration of Helsinki. Written informed consent was obtained from all participants when consenting to participate in the BASIC study, as well as when participating in the sub-study or undergoing elective caesarean section, prior to any testing.

### Sample collection and analytic procedure

Coded plasma samples were kept at room temperature for a maximum of one hour before being centrifuged for ten minutes in 1500 RCF (relative centrifugal force). The plasma was separated and stored at −70 °C before being sent to the Clinical Biomarker Facility at SciLife Lab, Uppsala, Sweden, for analysis. None of the samples used in this study had previously been thawed.

The samples were analysed for 92 protein biomarkers using Proseek Multiplex Inflammation I panel (Olink Bioscience, Uppsala, Sweden), which is based on the proximity extension assay (PEA) technology^42^. Limit of detection (LOD) was determined for each biomarker based on the mean value of triplicate negative controls analysed in each run. Data were presented in unit Normalized Protein Expression (NPX), obtained through normalising Cq-values against extension control, interplate control, and a correction factor. The NPX values correspond to relative quantification between samples and are presented on log2 scale. A list of the 92 inflammation markers analysed in the panel with corresponding UniProt identities is reported elsewhere^43^. The same batch of reagents was used for all samples, with cases and controls evenly distributed within the plates. Further details on the methodology have been described by Bränn *et al*.^31^.

Seventeen pregnancy samples and three postpartum samples were excluded due to technical reasons or missing data on exposure variables, which resulted in 321 samples analysed from pregnant women and 189 from postpartum women.

### Study variables

#### Inflammation summary variable

A summary variable was constructed in order to capture a particular woman’s overall level of immune system activation. This was done by combining information from all inflammatory markers with a detectable NPX value for more than 75% of the blood samples (pregnancy samples, n = 70; postpartum samples, n = 66), separately for the pregnancy and postpartum samples, and is a new approach used in a previous study by our research group^31^. Firstly, the NPX-value of every inflammatory marker was transformed to a Z-score according to the formula (value - mean)/SD. Secondly, a mean of those Z-scores was calculated for each participating woman. Finally, the resulting mean was transformed to a final Z-score, representing a summary inflammatory profile, the inflammation summary variable, for each woman.

#### Exposure variables and related variables

In order to address the main exposure variable, seasonality, a Lomb-Scargle Periodogram^44^ was used to identify if there was any periodicity in the data. The periodicity was investigated using day of sampling and the inflammation summary variable. Thereafter, sine and cosine functions were constructed as a measurements of seasonality based on a method by Stolwijk, Straatman, and Zielhuis^45^, using day of sampling. The sin and cosine function below were integrated in the regression models for both time periods:

- sin(2*pi*day of sampling/366)
- cos(2*pi*day of sampling/366)

Trigonometric relationships could then be used to transform the estimated coefficients into more intuitive measures such as amplitude, horizontal shift, and day of peak/trough.

Most of the background characteristics derived from the web surveys and were self-reported. At gestational week 17, the participants were asked about their height and weight, highest educational attainment, depression history, allergies, and whether the pregnancy was planned. Data on previous depression were derived from the question “Have you ever suffered from depression?”, with yes/no as alternative answers, combined with a question on whether the participant had ever visited a psychologist or psychiatrist. Information on allergies was derived from the question “Have you ever had any of these diseases before you became pregnant?”, where allergy was one alternative answer. Lastly, planned pregnancy derived from the question “Was the pregnancy planned”?, with yes/no as alternative answers. At gestational week 32, the participants were asked about current employment, hours of sleep per night, and they filled out the EPDS. At six weeks postpartum, data was collected on hours of sleep per night, breastfeeding, baby problems, perceived help with baby care by their partner, and stressful life events. Stressful life events were assessed using the Rosengren Scale^46^. In addition, the participants were asked to fill out the EPDS.

#### Outcome variables

The inflammation summary variable and the individual inflammatory markers were treated as outcome variables. A complete list of analysed inflammatory markers can be found in Supplementary TablesS1, S2. Due to a potential technical problem with the assessment of BDNF, reported by Olink Bioscience, this marker was excluded from the statistical analyses. Only inflammatory markers with a detectable NPX value for more than 75% of the blood samples were included. A complete list of excluded inflammatory markers is reported in Supplementary TableS3. Samples that had NPX values below LOD were replaced by LOD/sqrt(2)^47^. The values were expressed as log2(NPX).

#### Adjustments for oversampling

As previously mentioned, participants were more likely to be invited to the sub-study if they had an EPDS ≥12 (both time points) or were on treatment with SSRIs (pregnancy). Similarly, this study includes more elective caesarean sections than the general population, which were also oversampled based on depressive symptoms. Hence, in order to avoid bias, the models constructed for the pregnancy sample were adjusted for dichotomised EPDS, treatment with SSRIs, and elective caesarean sections. Similarly, the postpartum models were adjusted for dichotomised EPDS at 6 weeks postpartum.

### Statistical analyses

#### Exclusion criteria and adjustments

Decisions regarding further adjustments and exclusions were made based on the inflammation summary variable as outcome.

A confounder must be associated with both the exposure and the outcome; the following covariates were considered as potentially being associated with inflammation: time of sampling, body mass index (BMI), parity, planned pregnancy, and employment status at gestational week 32. Three linear regression models with the different covariates were created to determine if any of these covariates should be included in the final model. To assess time trends, the models also included year of sampling.

Model 1: oversampled variables + sine and cosine functions + year of sampling

Model 2: variables from model 1 and covariates with p < 0.2 (employment and planned pregnancy)

Model 3: variables from model 2 and all other possible covariates

The 10% change-in-estimator method was applied, meaning that if the covariates in Model 2 and 3 did not change the β coefficient of the sine and cosine functions from Model 1 by more than 10%, Model 1 was considered the final model^48^.

Once the final model was determined, we were concerned about women with preterm labour (n = 1), preeclampsia (n = 4), twin births (n = 5), inflammatory or rheumatoid disease (n = 6), smoking (n = 8), on-going or recently completed treatment with antibiotics, antivirals, or immunoglobulins (n = 8). Our concern was that these variables might be confounders or effect modifiers, however, due to their small numbers we would be unable to adjust for them appropriately. We therefore ran two models 1) including and 2) excluding these women, to see if the estimator was changed by more than 10%, i.e. to see if these women materially impacted our model estimates.

Decisions regarding adjustments and exclusions were based on the pregnancy inflammation summary variable. Based on the results from the 10% change-in-estimator method, the linear regression models were not adjusted nor were any participants excluded, hence, Model 1 was used.

#### Linear regression and bonferroni correction

For each individual inflammatory marker and the inflammation summary variable, a linear regression model was run. For each model the “seasonality” was assessed by performing a likelihood ratio test on the cos/sin variables simultaneously. These p-values were then corrected for multiple testing by using the Bonferroni correction.

To obtain more intuitive measures, we applied trigonometric relationships to the coefficients of the sine and cosine functions to calculate the amplitude (mean to extreme absolute difference of NPX), as well as when the peak and the trough occurred^45^.

β_1_ = coefficient sine curve

β _2_ = coefficient cosine curve

T = time period = 366 days

$$\begin{array}{ccc}Amplitude & = & \sqrt{\beta {1}^{2}+\beta {2}^{2}}\\ t & = & \arctan (\frac{\beta 1}{\beta 2})\times \frac{T}{2\pi }\end{array}$$

“If β_1_/β_2_ > 0, then *t* > 0 and indicates the first extreme; the other extreme value is found at *t* + *T*/2. If β_1_/β_2_ ≤ 0; the extreme values are found at *t* + *T/*2 and at *t* + *T*. If β_1_> 0, the first extreme is a maximum and the second a minimum; if β ≤ 0 it is the other way around^45^”. As the inflammatory markers were expressed on log2 scale, to obtain the expected relative difference between trough and peak, the following calculation was applied for each inflammatory marker: 100*(2^(2*amplitude)^−1).

#### Sub-analysis

As the focus of the BASIC study is peripartum mood and women with EPDS ≥12 were targeted when recruiting for the sub-study, there was an overrepresentation of participants suffering from depressive symptoms. This was the reason for adjusting for EPDS in the above mentioned analyses, but as a further step, a sub-analysis was conducted, including solely participants identified as not having depressive symptoms. The inclusion criteria were EPDS at blood sampling <13 (pregnancy) or <12 (postpartum), a negative outcome on the depression section of the M.I.N.I., as well as not reporting treatment with SSRIs, giving a sample size of 226 pregnancy samples and 131 postpartum samples. The cut-offs used for the EDPS score have been validated in a Swedish population^40,41^. The M.I.N.I. is a structured interview with the depression diagnosis corresponding to the DSM-V criteria for a depressive episode^49^. This interview was conducted at the research laboratory by researchers.

The statistical analyses were conducted in SPSS version 24 (IBM Corp, Armonk, NY) and R version 3.4^50^. Graphs were created in R. Statistical significance was set to *p*-value of <0.05, if not stated otherwise.

## Results

### Background characteristics

**Figure 1:**
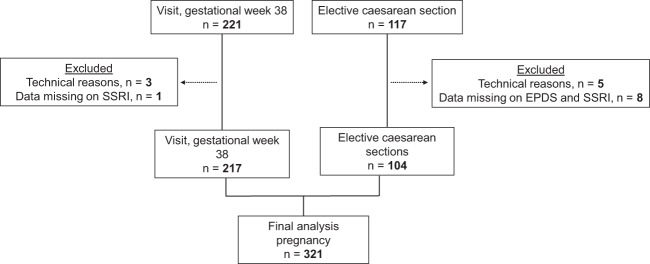
Flowchart of participating women in pregnancy.

**Figure 2:**
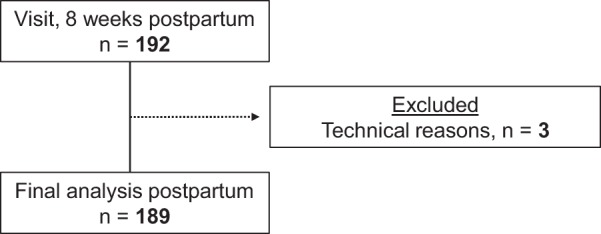
Flowchart of participating women in the postpartum period.

The background characteristics of the participants are listed in Table 1 and flowcharts of the participants can be seen in Figs 1 and 2. Briefly, for both pregnancy and postpartum, the median age was 32.0 years (interquartile range (IQR): 29.0–35.0). The median BMI was 23.3 kg/m2 (IQR: 21.3–26.1) in pregnancy and 23.0 (IQR: 21.1–25.3) postpartum. Most participants had a college or university degree and were working or studying at gestational week 32. Approximately 25% reported to have some type of allergy. In the postpartum group, 74% of the participants gave birth vaginally, and 75% breastfed exclusively. Lastly, in both groups, approximately one-third of the participants had depressive symptoms. Ten percent were on treatment with SSRIs at gestational week 32.

**Table 1:** Background characteristics of the study participants.

Characteristic	Pregnancy (n = 321) n (%)	Postpartum (n = 189) n (%)
Fasting sample (caesarean section)	104 (32.4)	0 (0.0)
Age (years), median (IQR)	32.0 (29.0–35.0)	32.0 (29.0–35.0)
BMI (kg/m^2^), median (IQR)	23.3 (21.3–26.1)	23.0 (21.1–25.3)
College/University	230 (74.7)	141 (79.2)
Current employment		
Full-time/part-time/studying	178 (66.9)	134 (75.7)
Unemployed/sick-leave/maternity leave	88 (33.1)	43 (24.3)
Smoking^a^	8 (2.5)	5 (2.6)
Allergy/ies	76 (24.8)	44 (24.4)
Rheumatoid or inflammatory disease	6 (1.9)	6 (3.2)
Antibiotics, antivirals, or immunoglobulins	9 (2.8)	3 (1.6)
Less than 6 hours of sleep per night	36 (12.8)	51 (27.1)
Primipara	117 (37.4)	94 (50.5)
Unplanned pregnancy	49 (18.0)	30 (16.9)
Preeclampsia	4 (1.2)	1 (0.5)
Days to or from childbirth, median (IQR)	18 (12.0–26.0)	69 (62.0–76.8)
Way of giving birth		
Spontaneous vaginal		140 (74.1)
Vacuum extraction		16 (8.5)
Elective caesarean		14 (7.4)
Emergency caesarean		16 (8.5)
Crash caesarean		3 (1.6)
Newborn gender = boy		98 (51.9)
Twins	5 (1.6)	2 (1.1)
Not exclusively breastfeeding		48 (25.5)
≥2 SLEs last 12 months		30 (16.0)
Previous depression	209 (67.6)	103 (57.2)
EPDS ≥12^b^	102 (31.8)	54 (28.6)
SSRI treatment^b^	32 (10.0)	
Sample month		
January	25 (7.8)	13 (6.9)
February	35 (10.9)	18 (9.5)
March	54 (16.8)	15 (7.9)
April	41 (12.8)	26 (13.8)
May	39 (12.1)	35 (18.5)
June	22 (6.9)	11 (5.8)
July	9 (2.8)	5 (2.6)
August	10 (3.1)	1 (0.5)
September	24 (7.5)	13 (6.9)
October	24 (7.5)	12 (6.3)
November	18 (5.6)	25 (13.2)
December	20 (6.2)	15 (7.9)

IQR = interquartile range BMI = body mass index; SLEs= stressful life events; EPDS = Edinburgh Postnatal Depression Scale; SSRI = selective serotonin reuptake inhibitor.

^a^At first contact with the maternity health care service during pregnancy, or postpartum (the latter only applicable to postpartum samples).

^b^Relevant due to oversampling.

### Periodicity test

The periodicity test (Lomb-Scargle) revealed a peak in the inflammation summary variable occurring annually (approximately every 366 days) in the pregnant study group (Supplementary Fig. S1). There was no seasonality in the postpartum inflammation summary variable (Supplementary Fig. S2).

### Main analysis

**Figure 3:**
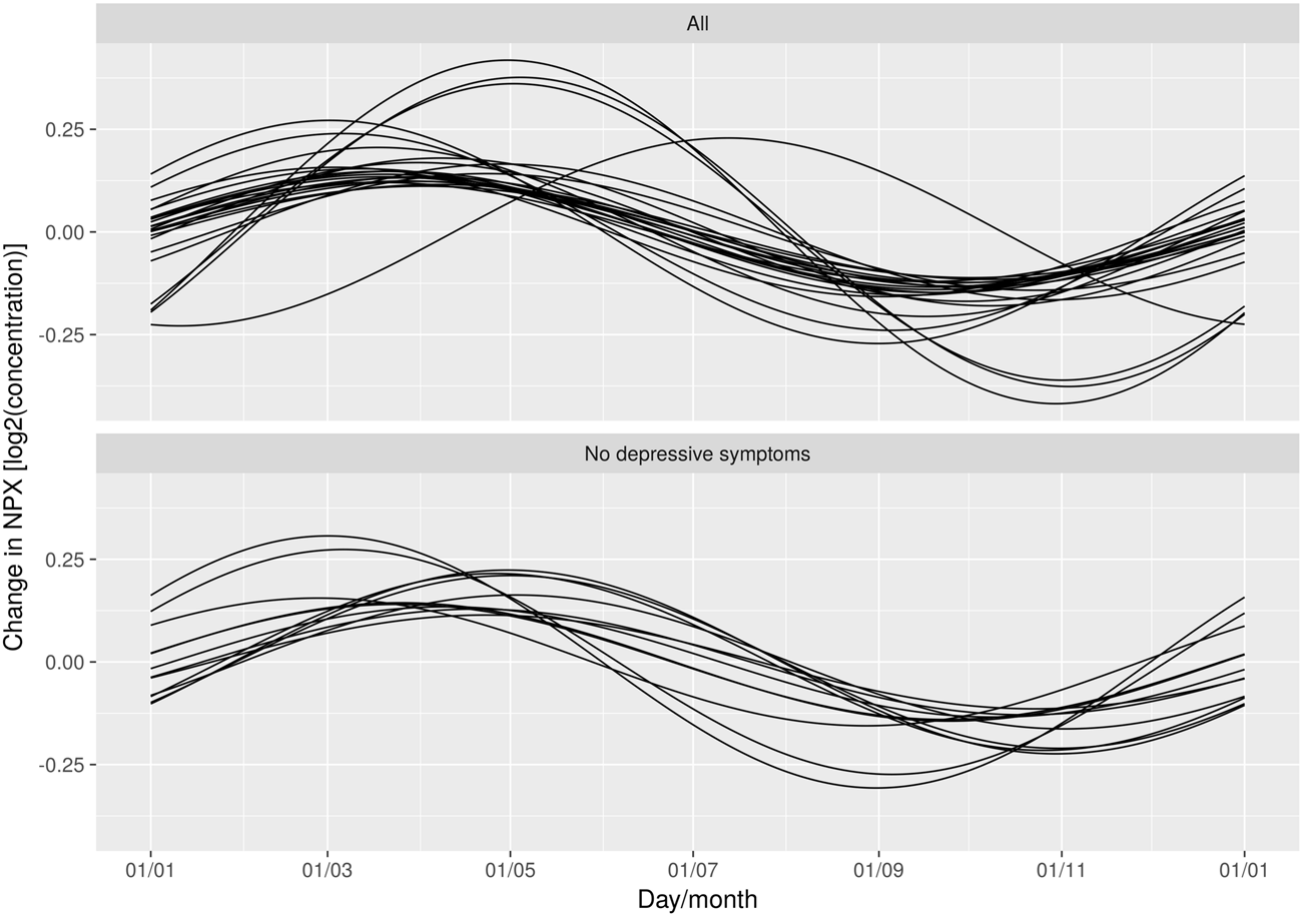
Graph of inflammatory markers exhibiting statistically significant seasonal variation after Bonferroni correction. The top graph illustrates 23 significant inflammatory markers found in the total sample. The bottom graph illustrates 11 inflammatory markers in pregnant women without depressive symptoms, as well as one postpartum marker.

**Figure 4:**
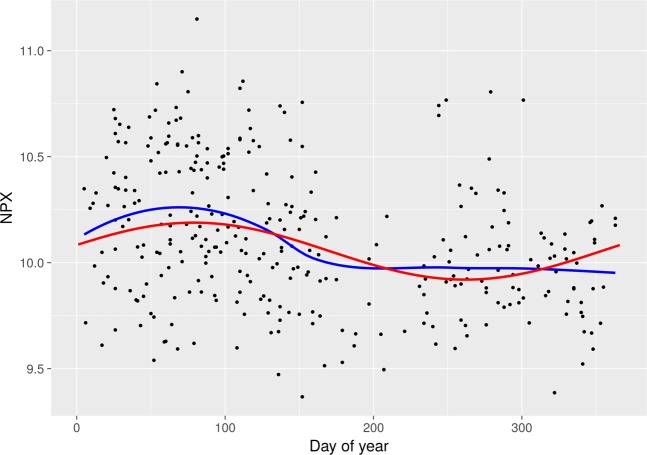
Scatter plot with NPX values of VEGF-A in relation to day of blood sampling in the pregnancy sample. Red line = cosine/sine fit from regression models; blue line = LOESS fit.

**Figure 5:**
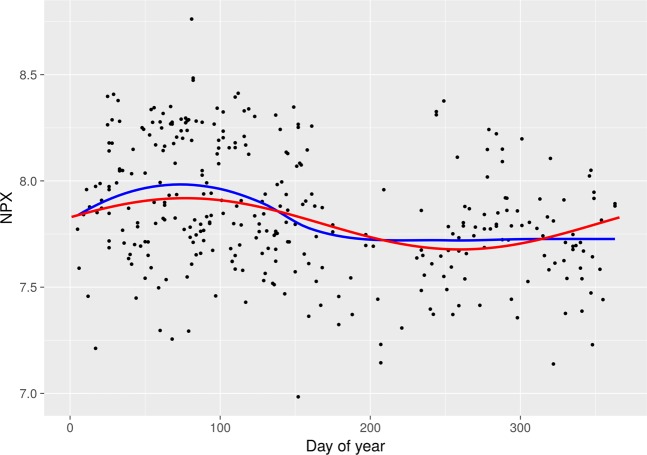
Scatter plot with NPX values of CSF-1 in relation to day of blood sampling in the pregnancy sample. Red line = cosine/sine fit from regression models; blue line = LOESS fit.

After Bonferroni correction, 23 inflammatory markers were found to follow a seasonal pattern during pregnancy (Table 2, Fig. 3). The peaks occurred between days 61 and 124 (March–May), while the day of trough varied from day 244 to 307 (August–November). The expected relative difference of inflammation between trough and peak varied between 16.7–78.6%. Of note, all inflammatory markers had peaks in spring and troughs in autumn. The inflammatory markers with statistically strongest seasonal associations were vascular endothelial growth factor A (VEGF-A) (p <0.001), macrophage colony-stimulating factor (CSF-1) (p <0.001), osteoprotegerin (OPG) (p <0.001), CUB domain-containing protein 1 (CDCP1) (p <0.001), and tumor necrosis factor receptor superfamily member 9 (TNFRSF9) (p <0.001). As an example of the raw data, scatter plots of VEGF-A and CSF-1 are shown in Figs 4 and 5. The inflammatory markers with the largest seasonal variation were STAM-binding protein (STAM-BP), axin-1 (AXIN1), and SIR2-like protein (SIRT2), with an expected relative difference of inflammation between trough and peak of 64.9%, 68.5%, and 78.6%, respectively.

**Table 2:** Seasonal variation of the significant inflammatory markers among pregnancy samples (n = 321) and postpartum samples (n = 189).

Inflammatory marker	Peak (day)	Trough (day)	Expected relative difference between trough and peak (%)^a^	Mean value	Bonferroni corrected p-value
*Pregnancy samples*					
VEGF-A	79	262	20.5	10.1	<0.001
OPG	77	260	33.0	10.5	<0.001
CSF-1	77	260	18.2	7.9	<0.001
CDCP1	65	248	39.4	2.7	<0.001
TNFRSF9	63	246	24.4	5.3	<0.001
CD244	113	296	21.8	5.2	0.001
Beta-NGF	91	274	18.6	0.8	0.001
MCP-2	61	244	45.7	7.5	0.001
CD5	92	275	20.5	3.0	0.002
STAM-BP	122	305	64.9	3.5	0.002
MCP-1	86	269	20.0	9.0	0.003
CD40	98	281	28.3	8.3	0.004
IL-15RA	92	275	16.7	0.2	0.005
SIRT2	120	303	78.6	3.7	0.005
IL-10RB	86	269	18.8	6.2	0.007
MCP-4	118	301	25.7	1.2	0.009
DNER	97	280	17.4	6.4	0.011
HGF	78	261	22.7	6.8	0.015
IL-12B	90	273	26.4	3.1	0.021
ARTN	83	266	22.7	0.8	0.022
IL-18R1	71	254	23.8	6.4	0.028
hGDNF	79	262	21.5	1.6	0.032
AXIN1	124	307	68.5	2.6	0.041
*Postpartum samples*					
MCP-4	194	11	37.3	1.8	0.045

^a^Change in inflammation level (not NPX value, see methods for more details).

VEGF-A: Vascular endothelial growth factor A; OPG: Osteoprotegerin; CSF-1: Macrophage colony-stimulating factor 1; CDCP1: CUB domain-containing protein 1; TNFRSF9: Tumor necrosis factor receptor superfamily member 9; CD244: Natural killer receptor 2B4; Beta-NGF: Beta-nerve growth factor; MCP-2: Monocyte chemotactic protein 2; CD5: T-cell surface glycoprotein CD5; STAM-BP: STAM-binding protein; MCP-1: Monocyte chemotactic protein 1; CD40: CD40L receptor; IL-15RA: Interleukin-15 receptor subunit alpha; SIRT2: SIR2-like protein 2; IL-10RB: Interleukin-10 receptor subunit beta; MCP-4: Monocyte chemotactic protein 4; DNER: Delta and Notch-like epidermal growth factor-related receptor; HGF: Hepatocyte growth factor receptor; IL-12B: Interleukin-12 receptor subunit beta; ARTN: Artemin; IL-18R1: Interleukin-18 receptor 1; hGDNF: Glial cell line-derived neurotrophic factor; AXIN1: Axin-1; NT-3: Neurotrophin-3.

One inflammatory marker, monocyte chemotactic protein 4 (MCP-4), was found to exhibit a seasonal pattern in the postpartum group (p = 0.045). MCP-4 peaked on day 194 (July) and had its trough on day 11 (January), with an expected relative difference of inflammation between trough and peak of 37.3% (Table 2, Fig. 3). Results from the linear regression for all inflammatory markers are reported in Supplementary Tables S1, S2.

### Sub-analysis

The sub-analysis included 226 pregnancy samples and 131 postpartum samples from participants without depressive symptoms. After Bonferroni correction, 11 inflammatory markers were significant in pregnancy samples (Fig. 3, Table 3). Among the postpartum samples, neurotrophin-3 (NT-3) had a peak on day 120, a trough on day 303, with an expected relative difference between trough and peak of 36.4% (p = 0.033) (Fig. 3, Table 3). Information on the seasonal pattern of all inflammatory markers is presented in Supplementary Tables S4, S5.

**Table 3:** Seasonal variation of significant inflammatory markers in pregnancy (n = 226) and postpartum (n = 131) samples among women without depressive symptoms.

Inflammatory marker	Peak (day)	Trough (day)	Expected relative difference between trough and peak (%)^a^	Mean value	Bonferroni corrected p-value
*Pregnancy samples*					
VEGF-A	84	267	22.1	10.2	0.000
CSF-1	84	267	21.5	7.9	0.000
CD244	123	306	25.3	5.2	0.002
MCP-4	121	304	33.9	1.3	0.002
MCP-2	60	243	53.0	7.5	0.005
TRANCE	116	299	34.8	2.1	0.012
IL-15RA	113	296	17.2	0.3	0.015
Beta-NGF	100	283	20.5	0.9	0.015
DNER	110	293	19.5	6.4	0.025
TNFRSF9	57	240	24.1	5.4	0.026
CCL19	65	248	46.2	8.3	0.044
*Postpartum samples*					
NT-3	120	303	36.4	1.7	0.033

^a^Change in inflammation level (not NPX value, see methods for more details).

VEGF-A: Vascular endothelial growth factor A; CSF1: Macrophage colony-stimulating factor 1; CD244: Natural killer receptor 2B4; MCP-4: Macrophage colony-stimulating factor 4; MCP-2: Macrophage colony-stimulating factor 2; TRANCE: Tumor necrosis factor ligand superfamily member 11; IL-15RA: Interleukin-15 receptor subunit alpha; Beta-NGF: Beta-nerve growth factor; DNER: Delta and Notch-like epidermal growth factor-related receptor; TNFRSF9: Tumor necrosis factor receptor superfamily member 9; CCL19: C-C motif chemokine 19; NT-3: Neutrotrophin-3.

## Discussion

The aim of this study was to investigate if there are seasonal variations in levels of peripheral inflammatory markers during late pregnancy and the early postpartum period. During pregnancy, an annually recurring pattern was found for 23 inflammatory markers and in the constructed inflammation summary variable. For the majority of the markers, the peak occurred in March–April and the trough in September–October. In the postpartum period, only one marker had a statistically significant seasonal pattern after Bonferroni correction.

The sub-analysis, which only included participants without depressive symptoms, revealed a seasonal pattern in 11 inflammatory markers, including one postpartum marker. Nine of the inflammatory markers from the pregnancy samples were among the 23 markers identified as having significant seasonality in the main analysis. There were small differences with regard to peak, trough, and the expected relative difference between trough and peak, when the results were compared with the main analysis. The reduction in number of inflammatory markers displaying seasonality could be the result of reduced power in detecting differences, as the sample sizes of these sub-groups (pregnancy and postpartum) were around 30% smaller. The similarities in results between the main and sub-analysis indicate that the seasonality was not driven by the inclusion of women with depressive symptoms. The low sample size of participants with depressive symptoms in this study would make the result of an analysis of seasonality in this subgroup dubious.

VEGF-A, OPG, and CSF-1 were found among the markers with the strongest statistical significance among pregnancy samples. VEGF-A is a growth factor and vasodilator involved in angiogenesis during pregnancy^51^, and inhibiting its effect has been associated with pregnancy complications such as gestational hypertension and preeclampsia^52^. Preeclampsia occurrence has been suggested to be increased among childbirths during the coldest months of the year^2^. It is theoretically plausible that seasonality of preeclampsia could be associated to the seasonal pattern of VEGF-A shown in the current study, as this marker has its trough in the end of September. OPG is a tumour necrosis factor decoy receptor that has been reported to vary in maternal plasma and serum concentrations throughout pregnancy, possibly having a role in the regulation of bone turnover during pregnancy^53^. Vitamin D has been reported as a bone resorption stimulating factor, which inhibits the production of OPG^54^. In Northern Europe, vitamin D levels are generally high in late summer and reach their minimum after winter^55^. Hence, seasonally varying levels of vitamin D may to some extent underlie the patterns observed in this marker. CSF-1, a chemokine with anti-inflammatory properties, has been suggested to play a role in shaping decidual macrophages in early pregnancy, and thus having a key role in supporting a tolerant immune milieu^56^. Levels of CSF-1 have been reported to increase in late pregnancy^57^. SIRT2 was the marker with the largest expected relative difference of inflammation between trough and peak. SIRT2 is an enzyme involved in stress response, and a reduction in SIRT2 has been reported in both preeclampsia^58^ and in pregnant women who later develop postpartum depressive symptoms^31^. Whether the seasonal pattern of SIRT2 could be implicated in the varying incidence of preeclampsia throughout the year remains to be investigated. No other studies on seasonality in this marker have been identified. The only inflammatory marker that displayed an annually recurring pattern among the postpartum samples was MCP-4, having its peak in the summer. MCP-4 is a chemokine that plays a role in the immune recruitment of monocytes and T lymphocytes^59^, and has been associated with allergic respiratory disease trough activation of histamine release^60^. MCP-4 also displayed significant variations in the pregnancy sample, with an earlier peak in spring. Regarding the seasonality identified in postpartum NT-3 levels, after the removal of participants with depressive symptoms, it could be speculated that women with depressive symptoms have lower seasonal fluctuations in comparison with healthy women. Theoretically, this could originate from a reduced capacity of the immune system of individuals with depressive symptoms to adapt or respond to outside factors. Further assessment of women with peripartum depressive symptoms would add knowledge on the potential association between seasonal peripartum depressive symptoms and fluctuations in inflammatory markers.

The fact that seasonality was only identified in pregnancy and not in the postpartum period contradicts our hypothesis and could be explained by sizable and rapid changes in the immune system after childbirth, as well as other factors such as wound healing, which is likely to affect the immune system to a larger degree than season. In mouse models, VEGF-A has been reported to dramatically drop postpartum following the rapid reduction in progesterone levels^51^. Similarly, at 12 weeks postpartum, OPG has been reported to not differ significantly from pre-conception levels^61^. Of note, in a study based on the same data, 15 out of the 23 inflammatory markers identified as having a significant seasonal variation among pregnancy samples in the present study, were significantly lower around 8 weeks postpartum compared with levels at gestational week 38^62^. In parallel, it has to be noted that there were more samples available for analysis from pregnant women, and that the significant rise in levels of most inflammatory markers during pregnancy might have rendered seasonal differences during pregnancy easier to detect.

Previous studies have reported on seasonal variations in levels of IL-6 and TNF-α^38^. In the current study, TNF-α was excluded based on the fact that too many samples were under LOD. These two markers are of particular interest to study with regard to preterm birth and preeclampsia. Prior to the Bonferroni correction, IL-6 was significantly different (p = 0.001) in pregnancy, exhibiting a similar seasonal pattern as the final significant inflammatory markers. IL-6 has been associated with preterm birth, a condition reported to increase during the summer months, although a winter peak also have been identified^5^. In this study, samples from premature births were too few to be studied and compared with term births, in terms of seasonal patterns.

Considering that gene expression related to the immune system has been reported to have both summer and winter peaks^34^, it is interesting that all inflammatory markers in the current study exhibited only a spring peak. Nevertheless, the same peak and trough pattern as in the current study has been reported in total white blood cell count, monocytes, and neutrophils^34^. Discrepancies in peaks and troughs between studies conducted on gene expression and those investigating circulating markers could be due to post-translational processes^38^.

The peak and trough pattern identified in the current study could be attributed to sunlight, presence of pollen, or diet. Relating back to vitamin D, the production of which is dependent on sunlight, a potential immunomodulatory role of this vitamin in the pathogenesis of preeclampsia has been described^63^. Vitamin D deficiency has also been implicated in the increased risk of immune-mediated diseases when being born during certain seasons^64–66^. Elevated levels of vitamin D in the summer have been associated with a reduced capacity of certain T cells to produce pro-inflammatory cytokines such as IL-2, IL-17, and IFN-γ^67^. A decrease in secretion of TNF and IL-6 is also associated with increased vitamin D levels^68^. With regards to the results of the current study, it is interesting to consider that the spring peak and autumn trough identified in inflammatory markers might emerge from a seasonal pattern in vitamin D. The spring peak could also represent remnants of an infection having occurred during winter. Furthermore, the spring peak also corresponds quite well with the start of the pollen season in Sweden, which triggers an inflammatory reaction in sensitive individuals^69,70^. As previously mentioned, around 25% of the participants in the current study reported to have some type of allergy. Immune related seasonal variations have been noted in human gut microbiota. In this case, variations in diet are proposed to be the mechanism behind the seasonal pattern^71^. Variation of the gut microbiota is a possible mechanism for changes in inflammatory status^72^. In the current sub-study there was no information on diet available.

The growing literature^73–75^ on the negative health aspects of being born in a specific season is interesting to consider from the point of possible epigenetic programming, and the possible role of seasonal variation in the immune system of a pregnant woman. A review of seasonal birth studies regarding different neurological diseases showed the most consistent pattern for epilepsy with an excess of births in winter, while MS, amyotrophic lateral sclerosis, and possibly Parkinson’s disease seem to be more common in spring births^75^. Studies of cerebral palsy are not conclusive, although there are suggestions that there may be an excess of summer births^75^. Similarly, the birth of patients with schizophrenia, recurrent depressive disorder, and bipolar affective disorder has been reported to be significantly higher in certain seasons^74^.

Whether a seasonal patterns in inflammatory markers can drive the proposed seasonality of some pregnancy complications warrants further investigations and data should preferably be derived from the same country, or countries with similar environmental cues. Even if no clear-cut evidence for the association between these seasonal patterns will be found, it can be hypothesized that it is not the seasonality of the inflammatory markers per se that results in pregnancy complications. Perhaps, among women with unfavourable levels of inflammatory markers, a seasonal peak or trough may result in a shift from a normal pregnancy to a complicated one. From an evolutionary perspective, seasonality has been crucial in generating biodiversity and has given rise to physiological adaptations and behaviour^76^. Thus, it could also be speculated that a seasonal pattern in inflammatory markers is a sign of an adaptive immune response and that problems occur when the system does not adapt to seasonal (or other) variations.

A major strength of the current study is its novelty, investigating seasonal variations in inflammatory markers in the peripartum period, adding to the literature on the seasonality of the immune system. Furthermore, it reports on a large number of inflammatory markers and includes a fairly large number of participants. The statistical methods, using sine and cosine functions can be seen as more robust in comparison with a regression model only using season, many times defined as quarters of the year, as exposure. Using sine and cosine functions pin-points the fluctuations with greater precision. In addition, in comparison with a number of other statistical tests for investigating seasonality, it allows for adjustments of confounders, if appropriate. A limitation pertains to the blood sampling taking place at the convenience of the participant; the time of sampling during the day was therefore not standardised. Nevertheless, the analyses for adjustments did not suggest that time of sampling had a significant impact on the inflammation summary variable. Furthermore, we acknowledge the underrepresentation of summer samples. A major difficulty when assessing women in the peripartum period is the dynamic changes in several bodily parameters. Multiple samples at different time-points in pregnancy and the postpartum period from the same woman would have been preferable, to facilitate taking into consideration intrapersonal variations throughout the peripartum period. However, this was not possible within the scope of this study. With regards to seasonality, it would have been ideal to follow the participants also in consecutive pregnancies, in order to distinguish between pregnancy-related and seasonality related changes.

As also discussed by Dopico *et al*.^34^ in their study on seasonal gene expression, understanding seasonality in inflammatory markers may be of clinical relevance as it could result in inter- and intra-person variability of the immune profile depending on when during the year the blood samples are collected.

In conclusion, seasonality in peripheral inflammatory markers was common in late pregnancy but not in the postpartum period. This study provides insight for the understanding of pregnancy complications including prematurity, preeclampsia, and peripartum depression where inflammation and alterations of the immune system could be of importance. Larger studies, using extensive inflammatory panels need to be conducted in order to replicate these results and provide further insight into the effect of season on the immune system in the peripartum setting. These results might also have important practical implications, and researchers might in the future need to consider and adjust for season when designing studies or interpreting results on inflammatory markers in the different fields of peripartum research.

## Supplementary information

Supplementary figures and tables

## Data Availability

The datasets generated during and/or analysed during the current study are available from the corresponding author on reasonable request.
