# Supplementary material for: Spring peaks and autumn troughs identified in peripheral inflammatory markers during the peripartum period
Source: Sci Rep. 2019 Oct 25;9:15328. doi: 10.1038/s41598-019-51527-9 (PMC6814733; doi:10.1038/s41598-019-51527-9)
Supplement: Supplementary file 1 — Supplementary figures and tables [file 41598_2019_51527_MOESM1_ESM.pdf]

Supplementary material

Spring peaks and autumn troughs identified in peripheral inflammatory markers during the peripartum period

Hanna E. Henriksson[1,*], Richard A. White[2], Stavros I. Iliadis[1], Emma Fransson[1,3], Fotios C. Papadopoulos[4], Inger Sundström-Poromaa[1], Alkistis Skalkidou[1]

[1] Department of Women's and Children's Health, Uppsala University, Uppsala, Sweden

[2] Norwegian Institute of Public Health, Oslo, Norway

[3] Department of Microbiology, Tumor and Cell Biology, Karolinska Institutet, Stockholm, Sweden

[4] Department of Neuroscience, Psychiatry, Uppsala University, Uppsala, Sweden

* Corresponding author

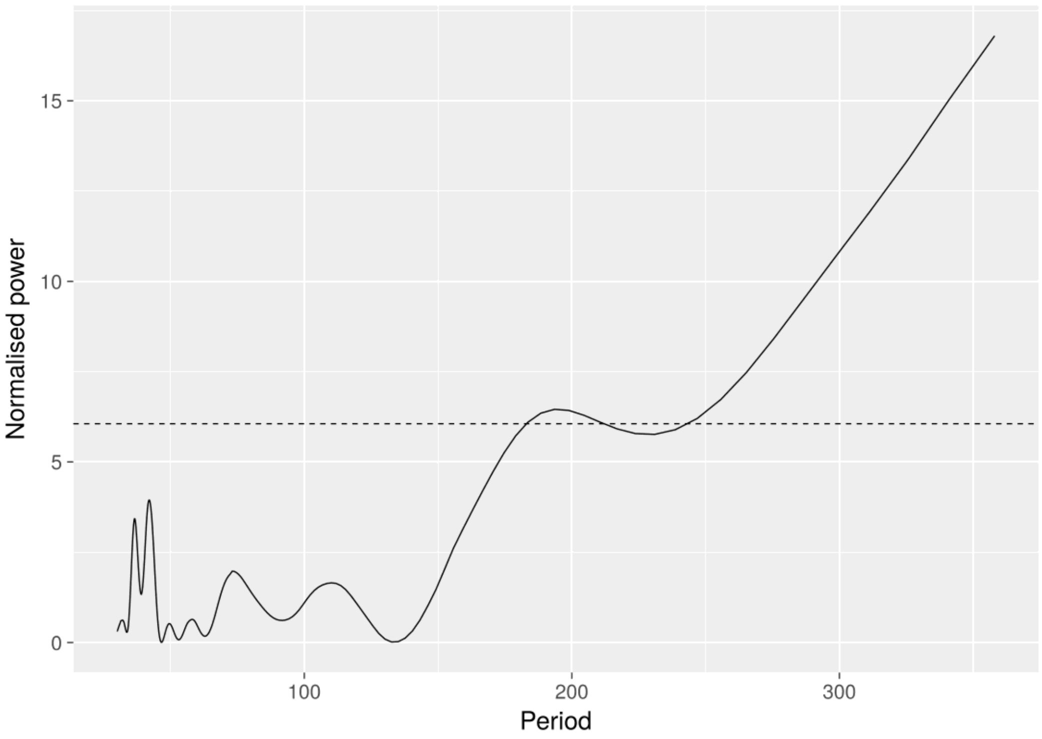

Supplementary Figure S1. Lomb-Scargle Periodogram for the pregnancy sample, indicating peak in the inflammation summary variable around day 366.

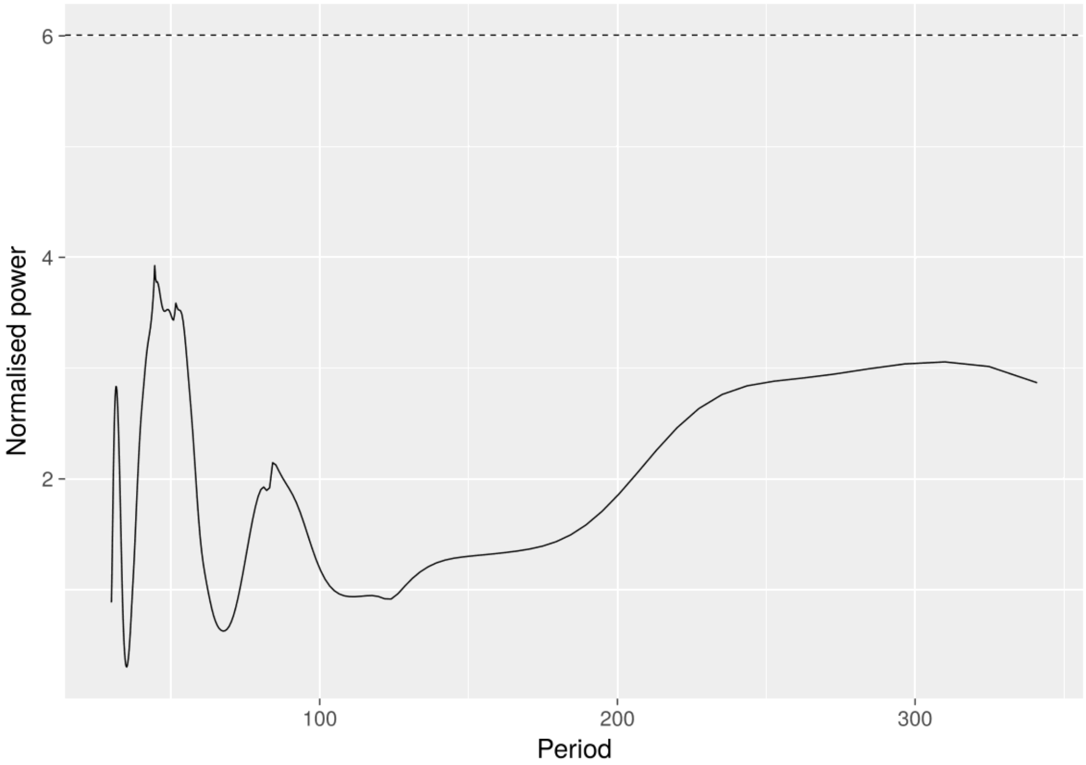

Supplementary Figure S2. Lomb-Scargle Periodogram for the postpartum sample, indicating no seasonality.

Supplementary Table S1. Linear regression derived β coefficient, standard error, 95% confidence interval (CI), as well as uncorrected and Bonferroni corrected *p*-values for 70 inflammatory markers and the inflammation summary variable in the pregnancy sample (n= 321).

Inflammatory marker	Variable	β coefficient	Standard error	95% CI	*p*-value	Overall *p*-value	Bonferroni corrected overall *p*-value
101_IL_8	sin366	0.150	0.044	0.064, 0.236	0.001	0.001	0.092
101_IL_8	cos366	0.077	0.051	-0.023, 0.177	0.132		
101_IL_8	year_trend	-0.082	0.031	-0.143, -0.021	0.009		
102_VEGF_A	sin366	0.131	0.020	0.092, 0.170	0.000	0.000	<0.001*
102_VEGF_A	cos366	0.029	0.023	-0.017, 0.074	0.216		
102_VEGF_A	year_trend	-0.126	0.014	-0.154, -0.099	0.000		
106_hGDNF	sin366	0.137	0.035	0.069, 0.206	0.000	0.000	0.032*
106_hGDNF	cos366	0.030	0.041	-0.050, 0.109	0.462		
106_hGDNF	year_trend	-0.137	0.025	-0.186, -0.088	0.000		
107_CDCP1	sin366	0.215	0.042	0.132, 0.298	0.000	0.000	<0.001*
107_CDCP1	cos366	0.105	0.049	0.009, 0.202	0.033		
107_CDCP1	year_trend	-0.140	0.030	-0.199, -0.081	0.000		
108_CD244	sin366	0.133	0.030	0.074, 0.191	0.000	0.000	0.001*
108_CD244	cos366	-0.051	0.035	-0.119, 0.017	0.140		
108_CD244	year_trend	-0.163	0.021	-0.204, -0.122	0.000		
109_IL_7	sin366	0.140	0.050	0.042, 0.238	0.005	0.009	0.630
109_IL_7	cos366	0.082	0.058	-0.031, 0.196	0.157		
109_IL_7	year_trend	-0.130	0.035	-0.199, -0.060	0.000		
110_OPG	sin366	0.199	0.040	0.120, 0.278	0.000	0.000	<0.001*
110_OPG	cos366	0.051	0.047	-0.041, 0.143	0.276		
110_OPG	year_trend	-0.107	0.029	-0.163, -0.051	0.000		
111_LAP.TGF_beta_1	sin366	0.082	0.039	0.006, 0.158	0.035	0.104	1.000
111_LAP.TGF_beta_1	cos366	0.016	0.045	-0.073, 0.104	0.729		
111_LAP.TGF_beta_1	year_trend	-0.159	0.028	-0.213, -0.105	0.000		
112_uPA	sin366	0.072	0.027	0.019, 0.125	0.008	0.025	1.000
112_uPA	cos366	0.019	0.031	-0.042, 0.081	0.533		
112_uPA	year_trend	-0.083	0.019	-0.121, -0.046	0.000		
113_IL_6	sin366	0.196	0.058	0.082, 0.310	0.001	0.001	0.072
113_IL_6	cos366	0.119	0.068	-0.013, 0.252	0.078		
113_IL_6	year_trend	-0.019	0.041	-0.100, 0.062	0.646		
114_IL_17C	sin366	0.103	0.043	0.018, 0.189	0.018	0.025	1.000
114_IL_17C	cos366	0.073	0.050	-0.026, 0.172	0.152		
114_IL_17C	year_trend	-0.149	0.031	-0.210, -0.089	0.000		
115_MCP_1	sin366	0.131	0.029	0.074, 0.188	0.000	0.000	0.003*
115_MCP_1	cos366	0.012	0.034	-0.054, 0.078	0.720		
115_MCP_1	year_trend	-0.078	0.021	-0.119, -0.038	0.000		
117_CXCL11	sin366	0.179	0.071	0.040, 0.318	0.012	0.002	0.119
117_CXCL11	cos366	0.221	0.082	0.059, 0.382	0.008		
117_CXCL11	year_trend	-0.180	0.050	-0.279, -0.081	0.000		
118_AXIN1	sin366	0.318	0.094	0.134, 0.503	0.001	0.001	0.041*
118_AXIN1	cos366	-0.201	0.109	-0.416, 0.014	0.067		
118_AXIN1	year_trend	-0.632	0.067	-0.763, -0.501	0.000		
120_TRAIL	sin366	0.085	0.027	0.033, 0.138	0.002	0.006	0.410
120_TRAIL	cos366	0.021	0.031	-0.040, 0.082	0.500		
120_TRAIL	year_trend	-0.140	0.019	-0.177, -0.103	0.000		
122_CXCL9	sin366	0.156	0.068	0.023, 0.289	0.022	0.002	0.174
122_CXCL9	cos366	0.215	0.079	0.060, 0.369	0.007		
122_CXCL9	year_trend	-0.182	0.048	-0.276, -0.087	0.000		
123_CST5	sin366	0.112	0.030	0.052, 0.171	0.000	0.001	0.089
123_CST5	cos366	0.018	0.035	-0.051, 0.087	0.612		

123_CST5	year_trend	-0.096	0.022	-0.138, -0.054	0.000		
126_OSM	sin366	0.168	0.073	0.024, 0.311	0.023	0.067	1.000
126_OSM	cos366	-0.030	0.085	-0.197, 0.137	0.723		
126_OSM	year_trend	-0.028	0.052	-0.130, 0.074	0.592		
128_CXCL1	sin366	0.146	0.064	0.021, 0.272	0.023	0.008	0.588
128_CXCL1	cos366	0.166	0.075	0.019, 0.312	0.027		
128_CXCL1	year_trend	-0.121	0.046	-0.210, -0.031	0.009		
130_CCL4	sin366	0.133	0.046	0.044, 0.222	0.004	0.002	0.129
130_CCL4	cos366	0.116	0.053	0.012, 0.220	0.029		
130_CCL4	year_trend	-0.080	0.032	-0.143, -0.017	0.014		
131_CD6	sin366	0.139	0.042	0.057, 0.220	0.001	0.002	0.134
131_CD6	cos366	-0.058	0.048	-0.153, 0.037	0.233		
131_CD6	year_trend	-0.110	0.030	-0.168, -0.052	0.000		
132_SCF	sin366	0.053	0.032	-0.010, 0.116	0.102	0.193	1.000
132_SCF	cos366	-0.027	0.037	-0.101, 0.046	0.466		
132_SCF	year_trend	-0.097	0.023	-0.142, -0.052	0.000		
133_IL_18	sin366	0.151	0.045	0.063, 0.240	0.001	0.004	0.274
133_IL_18	cos366	0.025	0.053	-0.078, 0.128	0.639		
133_IL_18	year_trend	-0.082	0.032	-0.145, -0.019	0.011		
135_TGFA	sin366	0.086	0.040	0.006, 0.165	0.035	0.020	1.000
135_TGFA	cos366	0.091	0.047	-0.001, 0.183	0.053		
135_TGFA	year_trend	-0.029	0.029	-0.085, 0.027	0.314		
136_MCP_4	sin366	0.148	0.038	0.074, 0.223	0.000	0.000	0.009*
136_MCP_4	cos366	-0.073	0.044	-0.160, 0.014	0.100		
136_MCP_4	year_trend	-0.068	0.027	-0.121, -0.015	0.013		
137_CCL11	sin366	0.093	0.039	0.015, 0.170	0.019	0.021	1.000
137_CCL11	cos366	0.074	0.046	-0.016, 0.163	0.109		
137_CCL11	year_trend	-0.113	0.028	-0.168, -0.058	0.000		
138_TNFSF14	sin366	0.152	0.042	0.069, 0.235	0.000	0.002	0.114
138_TNFSF14	cos366	-0.022	0.049	-0.118, 0.075	0.661		
138_TNFSF14	year_trend	-0.108	0.030	-0.167, -0.048	0.000		
139_FGF_23	sin366	0.132	0.086	-0.038, 0.301	0.128	0.185	1.000
139_FGF_23	cos366	-0.098	0.101	-0.295, 0.099	0.332		
139_FGF_23	year_trend	0.072	0.061	-0.049, 0.192	0.243		
141_FGF_5	sin366	0.089	0.032	0.027, 0.151	0.005	0.009	0.629
141_FGF_5	cos366	0.051	0.037	-0.021, 0.123	0.166		
141_FGF_5	year_trend	-0.077	0.022	-0.121, -0.033	0.001		
142_MMP_1	sin366	0.238	0.073	0.094, 0.381	0.001	0.005	0.334
142_MMP_1	cos366	0.065	0.085	-0.102, 0.232	0.449		
142_MMP_1	year_trend	-0.287	0.052	-0.389, -0.185	0.000		
143_LIF_R	sin366	0.101	0.041	0.020, 0.182	0.015	0.029	1.000
143_LIF_R	cos366	0.056	0.048	-0.038, 0.149	0.248		
143_LIF_R	year_trend	-0.135	0.029	-0.193, -0.078	0.000		
144_FGF_21	sin366	0.101	0.138	-0.170, 0.371	0.466	0.246	1.000
144_FGF_21	cos366	0.247	0.160	-0.067, 0.561	0.125		
144_FGF_21	year_trend	-0.080	0.098	-0.271, 0.112	0.417		
145_CCL19	sin366	0.211	0.058	0.096, 0.326	0.000	0.001	0.050
145_CCL19	cos366	0.101	0.068	-0.032, 0.234	0.138		
145_CCL19	year_trend	-0.131	0.042	-0.212, -0.049	0.002		
148_IL_15RA	sin366	0.111	0.025	0.062, 0.161	0.000	0.000	0.005*
148_IL_15RA	cos366	-0.001	0.029	-0.058, 0.056	0.972		
148_IL_15RA	year_trend	-0.089	0.018	-0.125, -0.054	0.000		
149_IL_10RB	sin366	0.123	0.028	0.068, 0.179	0.000	0.000	0.007*
149_IL_10RB	cos366	0.011	0.033	-0.053, 0.076	0.734		
149_IL_10RB	year_trend	-0.120	0.020	-0.159, -0.081	0.000		
151_IL_18R1	sin366	0.144	0.037	0.071, 0.218	0.000	0.000	0.028*
151_IL_18R1	cos366	0.053	0.044	-0.032, 0.138	0.226		
151_IL_18R1	year_trend	-0.146	0.027	-0.198, -0.094	0.000		

152_PD_L1	sin366	0.119	0.045	0.031, 0.207	0.008	0.017	1.000
152_PD_L1	cos366	0.060	0.052	-0.041, 0.162	0.246		
152_PD_L1	year_trend	-0.113	0.032	-0.175, -0.051	0.000		
153_Beta_NGF	sin366	0.123	0.025	0.074, 0.172	0.000	0.000	0.001*
153_Beta_NGF	cos366	0.001	0.029	-0.056, 0.058	0.961		
153_Beta_NGF	year_trend	-0.092	0.018	-0.127, -0.057	0.000		
154_CXCL5	sin366	0.142	0.103	-0.060, 0.344	0.170	0.366	1.000
154_CXCL5	cos366	0.049	0.120	-0.186, 0.284	0.682		
154_CXCL5	year_trend	-0.153	0.073	-0.297, -0.010	0.037		
155_TRANCE	sin366	0.144	0.042	0.062, 0.226	0.001	0.001	0.060
155_TRANCE	cos366	-0.073	0.049	-0.168, 0.023	0.138		
155_TRANCE	year_trend	-0.143	0.030	-0.202, -0.085	0.000		
156_HGF	sin366	0.144	0.035	0.075, 0.212	0.000	0.000	0.015*
156_HGF	cos366	0.033	0.041	-0.046, 0.113	0.415		
156_HGF	year_trend	-0.086	0.025	-0.135, -0.038	0.001		
157_IL_12B	sin366	0.169	0.041	0.088, 0.250	0.000	0.000	0.021*
157_IL_12B	cos366	0.004	0.048	-0.090, 0.098	0.935		
157_IL_12B	year_trend	-0.144	0.029	-0.202, -0.086	0.000		
160_ARTN	sin366	0.146	0.036	0.076, 0.217	0.000	0.000	0.022*
160_ARTN	cos366	0.022	0.042	-0.060, 0.104	0.601		
160_ARTN	year_trend	-0.094	0.026	-0.144, -0.044	0.000		
161_MMP_10	sin366	0.123	0.054	0.017, 0.230	0.024	0.009	0.662
161_MMP_10	cos366	0.137	0.063	0.013, 0.261	0.031		
161_MMP_10	year_trend	-0.103	0.039	-0.179, -0.028	0.008		
162_IL_10	sin366	0.094	0.050	-0.005, 0.192	0.063	0.023	1.000
162_IL_10	cos366	0.123	0.058	0.009, 0.237	0.036		
162_IL_10	year_trend	-0.109	0.036	-0.179, -0.039	0.002		
164_CCL23	sin366	0.110	0.038	0.036, 0.184	0.004	0.014	0.992
164_CCL23	cos366	0.026	0.044	-0.060, 0.112	0.561		
164_CCL23	year_trend	-0.107	0.027	-0.160, -0.055	0.000		
165_CD5	sin366	0.134	0.029	0.078, 0.191	0.000	0.000	0.002*
165_CD5	cos366	-0.001	0.034	-0.067, 0.065	0.975		
165_CD5	year_trend	-0.137	0.021	-0.177, -0.097	0.000		
166_MIP_1.alpha	sin366	0.162	0.044	0.075, 0.248	0.000	0.001	0.087
166_MIP_1.alpha	cos366	0.030	0.051	-0.070, 0.131	0.554		
166_MIP_1.alpha	year_trend	-0.120	0.031	-0.182, -0.059	0.000		
167_Flt3L	sin366	0.064	0.031	0.003, 0.124	0.039	0.089	1.000
167_Flt3L	cos366	0.030	0.036	-0.040, 0.100	0.401		
167_Flt3L	year_trend	-0.108	0.022	-0.151, -0.065	0.000		
168_CXCL6	sin366	0.213	0.057	0.100, 0.325	0.000	0.001	0.081
168_CXCL6	cos366	0.027	0.067	-0.103, 0.158	0.684		
168_CXCL6	year_trend	-0.197	0.041	-0.276, -0.117	0.000		
169_CXCL10	sin366	0.167	0.066	0.038, 0.295	0.012	0.002	0.145
169_CXCL10	cos366	0.197	0.076	0.048, 0.347	0.010		
169_CXCL10	year_trend	-0.123	0.047	-0.215, -0.032	0.009		
170_4E_BP1	sin366	0.189	0.081	0.030, 0.349	0.021	0.001	0.079
170_4E_BP1	cos366	-0.267	0.094	-0.452, -0.081	0.005		
170_4E_BP1	year_trend	-0.206	0.058	-0.319, -0.093	0.000		
172_SIRT2	sin366	0.369	0.093	0.186, 0.552	0.000	0.000	0.005*
172_SIRT2	cos366	-0.197	0.108	-0.409, 0.016	0.070		
172_SIRT2	year_trend	-0.370	0.066	-0.500, -0.240	0.000		
173_CCL28	sin366	0.161	0.064	0.034, 0.287	0.013	0.008	0.579
173_CCL28	cos366	0.148	0.075	0.002, 0.295	0.049		
173_CCL28	year_trend	-0.072	0.046	-0.161, 0.018	0.119		
174_DNER	sin366	0.115	0.027	0.062, 0.168	0.000	0.000	0.011*
174_DNER	cos366	-0.010	0.032	-0.073, 0.052	0.742		
174_DNER	year_trend	-0.109	0.019	-0.147, -0.072	0.000		
175_EN_RAGE	sin366	0.157	0.059	0.041, 0.272	0.008	0.028	1.000

175_EN_RAGE	cos366	-0.017	0.068	-0.151, 0.117	0.804		
175_EN_RAGE	year_trend	-0.046	0.042	-0.128, 0.035	0.267		
176_CD40	sin366	0.179	0.040	0.100, 0.257	0.000	0.000	0.004*
176_CD40	cos366	-0.020	0.047	-0.111, 0.072	0.672		
176_CD40	year_trend	-0.257	0.029	-0.313, -0.201	0.000		
179_FGF_19	sin366	0.121	0.072	-0.019, 0.261	0.092	0.056	1.000
179_FGF_19	cos366	0.148	0.083	-0.015, 0.311	0.076		
179_FGF_19	year_trend	-0.158	0.051	-0.258, -0.059	0.002		
183_MCP_2	sin366	0.235	0.054	0.129, 0.340	0.000	0.000	0.001*
183_MCP_2	cos366	0.137	0.062	0.014, 0.259	0.029		
183_MCP_2	year_trend	-0.091	0.038	-0.165, -0.016	0.018		
184_CASP_8	sin366	0.156	0.050	0.058, 0.254	0.002	0.003	0.222
184_CASP_8	cos366	-0.076	0.058	-0.191, 0.038	0.190		
184_CASP_8	year_trend	-0.136	0.036	-0.206, -0.067	0.000		
185_CCL25	sin366	0.120	0.051	0.021, 0.219	0.019	0.002	0.149
185_CCL25	cos366	0.160	0.059	0.045, 0.275	0.007		
185_CCL25	year_trend	-0.093	0.036	-0.164, -0.023	0.010		
186_CX3CL1	sin366	0.107	0.033	0.042, 0.171	0.001	0.001	0.068
186_CX3CL1	cos366	0.079	0.038	0.003, 0.154	0.041		
186_CX3CL1	year_trend	-0.154	0.023	-0.200, -0.109	0.000		
187_TNFRSF9	sin366	0.139	0.029	0.081, 0.196	0.000	0.000	<0.001*
187_TNFRSF9	cos366	0.075	0.034	0.008, 0.142	0.030		
187_TNFRSF9	year_trend	-0.141	0.021	-0.182, -0.101	0.000		
188_NT_3	sin366	0.192	0.054	0.087, 0.298	0.000	0.001	0.102
188_NT_3	cos366	0.059	0.063	-0.064, 0.182	0.346		
188_NT_3	year_trend	-0.059	0.038	-0.134, 0.016	0.122		
189_TWEAK	sin366	0.084	0.027	0.030, 0.138	0.002	0.009	0.625
189_TWEAK	cos366	-0.010	0.032	-0.072, 0.052	0.753		
189_TWEAK	year_trend	-0.073	0.019	-0.111, -0.035	0.000		
190_CCL20	sin366	0.231	0.067	0.100, 0.362	0.001	0.002	0.157
190_CCL20	cos366	0.064	0.078	-0.088, 0.216	0.411		
190_CCL20	year_trend	-0.102	0.047	-0.195, -0.009	0.032		
192_STAMBP	sin366	0.312	0.077	0.162, 0.463	0.000	0.000	0.002*
192_STAMBP	cos366	-0.181	0.089	-0.356, -0.006	0.043		
192_STAMBP	year_trend	-0.377	0.054	-0.483, -0.270	0.000		
194_ADA	sin366	0.111	0.044	0.026, 0.197	0.011	0.030	1.000
194_ADA	cos366	-0.034	0.051	-0.134, 0.066	0.507		
194_ADA	year_trend	-0.124	0.031	-0.184, -0.063	0.000		
195_TNFB	sin366	0.096	0.033	0.031, 0.160	0.004	0.008	0.577
195_TNFB	cos366	-0.041	0.038	-0.116, 0.034	0.289		
195_TNFB	year_trend	-0.150	0.023	-0.196, -0.104	0.000		
196_CSF_1	sin366	0.117	0.018	0.081, 0.153	0.000	0.000	<0.001*
196_CSF_1	cos366	0.030	0.021	-0.012, 0.072	0.157		
196_CSF_1	year_trend	-0.090	0.013	-0.115, -0.064	0.000		
Summary variable	sin366	0.402	0.062	0.281, 0.524	0.000	0.000	<0.001*
Summary variable	cos366	0.087	0.072	-0.054, 0.228	0.228		
Summary variable	year_trend	-0.365	0.044	-0.451, -0.279	0.000		

* Bonferroni corrected *p*-value < 0.05

CI: confidence interval

Supplementary Table S2. Linear regression derived β coefficient, standard error, 95% confidence interval (CI), as well as uncorrected and Bonferroni corrected p-values for 66 inflammatory markers and the inflammation summary variable in the postpartum sample (n= 189).

Inflammatory marker	Variable	β coefficient	Standard error	95% CI	*p*-value	Overall *p*-value	Bonferroni corrected overall *p*-value
101_IL_8	sin366	0.032	0.078	-0.121, 0.186	0.681	0.314	1.000
101_IL_8	cos366	-0.099	0.086	-0.268, 0.071	0.256		
101_IL_8	year_trend	-0.018	0.055	-0.126, 0.090	0.741		
102_VEGF_A	sin366	-0.003	0.039	-0.079, 0.072	0.930	0.118	1.000
102_VEGF_A	cos366	-0.081	0.043	-0.164, 0.002	0.058		
102_VEGF_A	year_trend	0.033	0.027	-0.020, 0.086	0.226		
107_CDCP1	sin366	0.055	0.047	-0.038, 0.147	0.247	0.389	1.000
107_CDCP1	cos366	0.061	0.052	-0.041, 0.163	0.241		
107_CDCP1	year_trend	-0.077	0.033	-0.142, -0.012	0.022		
108_CD244	sin366	0.019	0.044	-0.068, 0.106	0.667	0.726	1.000
108_CD244	cos366	0.039	0.049	-0.057, 0.135	0.428		
108_CD244	year_trend	-0.011	0.031	-0.072, 0.050	0.727		
109_IL_7	sin366	-0.010	0.066	-0.139, 0.120	0.884	0.559	1.000
109_IL_7	cos366	-0.075	0.073	-0.217, 0.068	0.307		
109_IL_7	year_trend	-0.043	0.046	-0.134, 0.048	0.354		
110_OPG	sin366	0.046	0.039	-0.030, 0.122	0.237	0.322	1.000
110_OPG	cos366	0.058	0.043	-0.026, 0.142	0.175		
110_OPG	year_trend	-0.030	0.027	-0.083, 0.023	0.274		
111_LAP.TGF_beta_1	sin366	-0.041	0.045	-0.130, 0.047	0.363	0.021	1.000
111_LAP.TGF_beta_1	cos366	-0.138	0.050	-0.236, -0.041	0.006		
111_LAP.TGF_beta_1	year_trend	0.077	0.032	0.015, 0.139	0.015		
112_uPA	sin366	0.014	0.029	-0.043, 0.071	0.633	0.885	1.000
112_uPA	cos366	0.003	0.032	-0.060, 0.067	0.920		
112_uPA	year_trend	-0.019	0.021	-0.059, 0.021	0.355		
113_IL_6	sin366	-0.058	0.084	-0.223, 0.107	0.490	0.681	1.000
113_IL_6	cos366	0.017	0.093	-0.165, 0.199	0.855		
113_IL_6	year_trend	-0.030	0.059	-0.145, 0.086	0.617		
115_MCP_1	sin366	-0.011	0.039	-0.087, 0.066	0.784	0.101	1.000
115_MCP_1	cos366	-0.088	0.043	-0.172, -0.004	0.043		
115_MCP_1	year_trend	0.001	0.027	-0.053, 0.054	0.977		
117_CXCL11	sin366	0.024	0.099	-0.169, 0.217	0.807	0.029	1.000
117_CXCL11	cos366	-0.250	0.109	-0.463, -0.037	0.023		
117_CXCL11	year_trend	-0.012	0.069	-0.148, 0.123	0.857		
118_AXIN1	sin366	0.131	0.151	-0.166, 0.427	0.389	0.093	1.000
118_AXIN1	cos366	-0.238	0.167	-0.565, 0.088	0.155		
118_AXIN1	year_trend	0.053	0.106	-0.155, 0.261	0.620		
120_TRAIL	sin366	-0.044	0.028	-0.099, 0.012	0.125	0.290	1.000
120_TRAIL	cos366	-0.031	0.031	-0.092, 0.030	0.327		
120_TRAIL	year_trend	0.017	0.020	-0.022, 0.056	0.394		
122_CXCL9	sin366	0.124	0.097	-0.065, 0.314	0.200	0.281	1.000
122_CXCL9	cos366	0.152	0.107	-0.058, 0.361	0.157		
122_CXCL9	year_trend	-0.063	0.068	-0.196, 0.070	0.355		
123_CST5	sin366	0.024	0.054	-0.082, 0.130	0.653	0.612	1.000
123_CST5	cos366	-0.036	0.060	-0.153, 0.081	0.551		
123_CST5	year_trend	0.037	0.038	-0.037, 0.111	0.332		
126_OSM	sin366	-0.302	0.095	-0.487, -0.116	0.002	0.007	0.483
126_OSM	cos366	-0.136	0.105	-0.341, 0.068	0.194		
126_OSM	year_trend	0.078	0.067	-0.052, 0.209	0.241		
128_CXCL1	sin366	0.038	0.101	-0.161, 0.236	0.712	0.120	1.000

128_CXCL1	cos366	-0.187	0.112	-0.406, 0.033	0.097		
128_CXCL1	year_trend	0.017	0.071	-0.123, 0.156	0.817		
130_CCL4	sin366	0.042	0.063	-0.081, 0.165	0.503	0.356	1.000
130_CCL4	cos366	-0.059	0.069	-0.194, 0.076	0.396		
130_CCL4	year_trend	-0.007	0.044	-0.093, 0.079	0.868		
131_CD6	sin366	-0.023	0.058	-0.137, 0.091	0.691	0.745	1.000
131_CD6	cos366	-0.049	0.064	-0.174, 0.076	0.445		
131_CD6	year_trend	0.031	0.041	-0.049, 0.111	0.444		
132_SCF	sin366	0.050	0.036	-0.021, 0.121	0.172	0.388	1.000
132_SCF	cos366	0.019	0.040	-0.060, 0.097	0.644		
132_SCF	year_trend	-0.010	0.026	-0.060, 0.040	0.692		
133_IL_18	sin366	-0.059	0.063	-0.182, 0.064	0.349	0.533	1.000
133_IL_18	cos366	-0.067	0.069	-0.202, 0.069	0.335		
133_IL_18	year_trend	0.006	0.044	-0.080, 0.092	0.895		
134_SLAMF1	sin366	-0.145	0.064	-0.271, -0.018	0.026	0.075	1.000
134_SLAMF1	cos366	-0.099	0.071	-0.238, 0.040	0.163		
134_SLAMF1	year_trend	0.030	0.045	-0.059, 0.119	0.508		
135_TGFA	sin366	-0.069	0.037	-0.142, 0.004	0.064	0.140	1.000
135_TGFA	cos366	-0.060	0.041	-0.140, 0.020	0.146		
135_TGFA	year_trend	0.052	0.026	0.001, 0.103	0.047		
136_MCP_4	sin366	-0.042	0.054	-0.148, 0.064	0.443	0.001	0.045*
136_MCP_4	cos366	-0.225	0.060	-0.342, -0.108	0.000		
136_MCP_4	year_trend	0.051	0.038	-0.023, 0.126	0.177		
137_CCL11	sin366	0.076	0.055	-0.032, 0.185	0.171	0.306	1.000
137_CCL11	cos366	-0.002	0.061	-0.121, 0.118	0.979		
137_CCL11	year_trend	-0.025	0.039	-0.101, 0.051	0.525		
138_TNFSF14	sin366	-0.050	0.060	-0.167, 0.068	0.408	0.027	1.000
138_TNFSF14	cos366	-0.178	0.066	-0.307, -0.048	0.008		
138_TNFSF14	year_trend	-0.006	0.042	-0.089, 0.076	0.878		
139_FGF_23	sin366	0.005	0.057	-0.106, 0.117	0.924	0.992	1.000
139_FGF_23	cos366	0.008	0.063	-0.115, 0.130	0.905		
139_FGF_23	year_trend	-0.003	0.040	-0.081, 0.075	0.943		
141_FGF_5	sin366	-0.002	0.049	-0.097, 0.093	0.966	0.769	1.000
141_FGF_5	cos366	0.034	0.054	-0.071, 0.139	0.529		
141_FGF_5	year_trend	0.019	0.034	-0.048, 0.086	0.575		
142_MMP_1	sin366	0.145	0.100	-0.052, 0.341	0.150	0.034	1.000
142_MMP_1	cos366	-0.148	0.110	-0.365, 0.069	0.182		
142_MMP_1	year_trend	-0.138	0.070	-0.276, -0.001	0.050		
143_LIF_R	sin366	0.050	0.033	-0.015, 0.116	0.136	0.215	1.000
143_LIF_R	cos366	0.055	0.037	-0.017, 0.127	0.138		
143_LIF_R	year_trend	-0.039	0.024	-0.085, 0.007	0.099		
144_FGF_21	sin366	0.086	0.153	-0.213, 0.385	0.574	0.487	1.000
144_FGF_21	cos366	0.202	0.168	-0.128, 0.532	0.231		
144_FGF_21	year_trend	-0.054	0.107	-0.264, 0.156	0.616		
145_CCL19	sin366	0.017	0.084	-0.148, 0.182	0.839	0.740	1.000
145_CCL19	cos366	-0.054	0.093	-0.236, 0.128	0.560		
145_CCL19	year_trend	-0.011	0.059	-0.127, 0.105	0.856		
149_IL_10RB	sin366	-0.093	0.040	-0.171, -0.015	0.020	0.067	1.000
149_IL_10RB	cos366	-0.044	0.044	-0.130, 0.042	0.317		
149_IL_10RB	year_trend	0.016	0.028	-0.039, 0.071	0.566		
151_IL_18R1	sin366	0.038	0.051	-0.061, 0.138	0.448	0.219	1.000
151_IL_18R1	cos366	-0.060	0.056	-0.170, 0.049	0.281		
151_IL_18R1	year_trend	0.013	0.036	-0.056, 0.083	0.708		
153_Beta_NGF	sin366	0.006	0.030	-0.053, 0.066	0.841	0.799	1.000
153_Beta_NGF	cos366	0.022	0.034	-0.043, 0.088	0.508		
153_Beta_NGF	year_trend	-0.012	0.021	-0.054, 0.030	0.582		
154_CXCL5	sin366	0.224	0.153	-0.077, 0.525	0.146	0.169	1.000
154_CXCL5	cos366	-0.074	0.169	-0.406, 0.257	0.661		

154_CXCL5	year_trend	-0.007	0.108	-0.218, 0.204	0.947		
155_TRANCE	sin366	0.011	0.067	-0.120, 0.142	0.874	0.604	1.000
155_TRANCE	cos366	-0.061	0.074	-0.205, 0.084	0.412		
155_TRANCE	year_trend	-0.004	0.047	-0.096, 0.088	0.938		
156_HGF	sin366	-0.068	0.041	-0.148, 0.011	0.095	0.227	1.000
156_HGF	cos366	-0.017	0.045	-0.105, 0.071	0.706		
156_HGF	year_trend	0.027	0.028	-0.028, 0.083	0.336		
157_IL_12B	sin366	-0.048	0.065	-0.175, 0.079	0.459	0.467	1.000
157_IL_12B	cos366	-0.087	0.071	-0.226, 0.053	0.226		
157_IL_12B	year_trend	-0.013	0.045	-0.102, 0.075	0.767		
161_MMP_10	sin366	0.037	0.093	-0.146, 0.220	0.692	0.829	1.000
161_MMP_10	cos366	-0.025	0.103	-0.227, 0.177	0.806		
161_MMP_10	year_trend	-0.002	0.066	-0.130, 0.127	0.979		
162_IL_10	sin366	-0.101	0.069	-0.237, 0.034	0.146	0.052	1.000
162_IL_10	cos366	0.086	0.076	-0.064, 0.235	0.263		
162_IL_10	year_trend	0.007	0.049	-0.088, 0.102	0.886		
164_CCL23	sin366	-0.025	0.050	-0.124, 0.073	0.614	0.628	1.000
164_CCL23	cos366	0.029	0.055	-0.080, 0.137	0.605		
164_CCL23	year_trend	0.049	0.035	-0.020, 0.118	0.169		
165_CD5	sin366	-0.092	0.039	-0.169, -0.016	0.019	0.030	1.000
165_CD5	cos366	-0.093	0.043	-0.177, -0.009	0.032		
165_CD5	year_trend	0.054	0.027	0.001, 0.108	0.049		
166_MIP_1.alpha	sin366	0.004	0.048	-0.089, 0.098	0.928	0.324	1.000
166_MIP_1.alpha	cos366	-0.069	0.053	-0.172, 0.034	0.192		
166_MIP_1.alpha	year_trend	0.047	0.033	-0.018, 0.113	0.159		
167_Flt3L	sin366	-0.011	0.048	-0.105, 0.082	0.812	0.626	1.000
167_Flt3L	cos366	-0.050	0.053	-0.153, 0.053	0.344		
167_Flt3L	year_trend	-0.000	0.034	-0.066, 0.066	0.994		
168_CXCL6	sin366	-0.021	0.089	-0.195, 0.153	0.817	0.027	1.000
168_CXCL6	cos366	-0.248	0.098	-0.440, -0.056	0.012		
168_CXCL6	year_trend	0.053	0.062	-0.069, 0.175	0.396		
169_CXCL10	sin366	-0.039	0.109	-0.252, 0.174	0.719	0.814	1.000
169_CXCL10	cos366	0.038	0.120	-0.197, 0.273	0.750		
169_CXCL10	year_trend	-0.030	0.076	-0.180, 0.120	0.694		
170_4E_BP1	sin366	-0.059	0.103	-0.262, 0.143	0.565	0.039	1.000
170_4E_BP1	cos366	-0.285	0.114	-0.508, -0.062	0.013		
170_4E_BP1	year_trend	-0.097	0.072	-0.239, 0.045	0.181		
172_SIRT2	sin366	0.010	0.141	-0.266, 0.286	0.943	0.019	1.000
172_SIRT2	cos366	-0.392	0.155	-0.696, -0.088	0.012		
172_SIRT2	year_trend	0.088	0.099	-0.105, 0.282	0.374		
173_CCL28	sin366	0.018	0.059	-0.097, 0.134	0.755	0.901	1.000
173_CCL28	cos366	-0.011	0.065	-0.138, 0.117	0.871		
173_CCL28	year_trend	-0.084	0.041	-0.165, -0.003	0.043		
174_DNER	sin366	0.014	0.027	-0.039, 0.067	0.602	0.788	1.000
174_DNER	cos366	0.019	0.030	-0.039, 0.077	0.526		
174_DNER	year_trend	-0.031	0.019	-0.068, 0.007	0.109		
175_EN_RAGE	sin366	-0.143	0.070	-0.281, -0.005	0.043	0.045	1.000
175_EN_RAGE	cos366	-0.172	0.078	-0.324, -0.019	0.028		
175_EN_RAGE	year_trend	0.003	0.049	-0.093, 0.100	0.945		
176_CD40	sin366	0.044	0.054	-0.062, 0.150	0.416	0.178	1.000
176_CD40	cos366	-0.069	0.060	-0.185, 0.048	0.252		
176_CD40	year_trend	0.012	0.038	-0.062, 0.086	0.751		
179_FGF_19	sin366	-0.104	0.108	-0.316, 0.107	0.335	0.286	1.000
179_FGF_19	cos366	-0.185	0.119	-0.419, 0.048	0.122		
179_FGF_19	year_trend	0.065	0.076	-0.084, 0.213	0.395		
183_MCP_2	sin366	-0.006	0.089	-0.181, 0.169	0.949	0.178	1.000
183_MCP_2	cos366	-0.168	0.099	-0.361, 0.025	0.090		
183_MCP_2	year_trend	0.041	0.063	-0.082, 0.164	0.514		

184_CASP_8	sin366	0.020	0.069	-0.116, 0.155	0.777	0.008	0.508
184_CASP_8	cos366	-0.206	0.076	-0.356, -0.057	0.007		
184_CASP_8	year_trend	0.031	0.049	-0.064, 0.126	0.529		
185_CCL25	sin366	0.126	0.068	-0.008, 0.259	0.066	0.184	1.000
185_CCL25	cos366	0.065	0.075	-0.082, 0.212	0.385		
185_CCL25	year_trend	-0.056	0.048	-0.150, 0.038	0.242		
186_CX3CL1	sin366	-0.035	0.042	-0.118, 0.047	0.402	0.646	1.000
186_CX3CL1	cos366	-0.034	0.046	-0.125, 0.056	0.461		
186_CX3CL1	year_trend	0.012	0.029	-0.046, 0.069	0.695		
187_TNFRSF9	sin366	-0.004	0.040	-0.083, 0.075	0.923	0.556	1.000
187_TNFRSF9	cos366	-0.045	0.045	-0.133, 0.042	0.312		
187_TNFRSF9	year_trend	0.023	0.028	-0.033, 0.078	0.427		
188_NT_3	sin366	0.080	0.061	-0.040, 0.200	0.195	0.005	0.304
188_NT_3	cos366	-0.148	0.068	-0.280, -0.015	0.030		
188_NT_3	year_trend	0.287	0.043	0.202, 0.371	0.000		
189_TWEAK	sin366	0.004	0.035	-0.065, 0.073	0.907	0.837	1.000
189_TWEAK	cos366	-0.018	0.039	-0.095, 0.058	0.635		
189_TWEAK	year_trend	-0.010	0.025	-0.059, 0.038	0.682		
190_CCL20	sin366	0.185	0.098	-0.007, 0.377	0.060	0.169	1.000
190_CCL20	cos366	0.077	0.108	-0.134, 0.289	0.474		
190_CCL20	year_trend	-0.024	0.069	-0.158, 0.111	0.729		
192_STAMBP	sin366	0.075	0.114	-0.148, 0.299	0.510	0.007	0.453
192_STAMBP	cos366	-0.317	0.126	-0.564, -0.071	0.012		
192_STAMBP	year_trend	0.012	0.080	-0.145, 0.168	0.885		
194_ADA	sin366	0.032	0.060	-0.085, 0.149	0.592	0.083	1.000
194_ADA	cos366	-0.113	0.066	-0.242, 0.015	0.086		
194_ADA	year_trend	-0.023	0.042	-0.105, 0.059	0.579		
195_TNFB	sin366	-0.007	0.051	-0.106, 0.092	0.891	0.990	1.000
195_TNFB	cos366	-0.005	0.056	-0.115, 0.104	0.924		
195_TNFB	year_trend	-0.021	0.036	-0.090, 0.049	0.558		
196_CSF_1	sin366	-0.011	0.027	-0.064, 0.042	0.685	0.433	1.000
196_CSF_1	cos366	-0.038	0.030	-0.096, 0.020	0.201		
196_CSF_1	year_trend	-0.010	0.019	-0.047, 0.027	0.595		
Summary variable	sin366	0.062	0.113	-0.161, 0.284	0.586	0.182	1.000
Summary variable	cos366	-0.169	0.125	-0.415, 0.076	0.178		
Summary variable	year_trend	-0.001	0.080	-0.157, 0.155	0.991		

* Bonferroni corrected *p*-value < 0.05
CI: confidence interval

Supplementary Table S3. List of excluded inflammatory markers due to more than 25% of markers < limit of detection (LOD).

Pregnancy		Postpartum	
IL-1 alpha	Interleukin-1-alpha	IL-1 alpha	Interleukin-1-alpha
IL-2	Interleukin-2	IL-2	Interleukin-2
IL-2RB	Interleukin-2 receptor subunit beta	IL-2RB	Interleukin-2 receptor subunit beta
IL-4	Interleukin-4	IL-4	Interleukin-4
IL-5	Interleukin-5	IL-5	Interleukin-5
IL-10RA	Interleukin-10 receptor subunit alpha	IL-10RA	Interleukin-10 receptor subunit alpha
IL-13	Interleukin-13	IL-13	Interleukin-13
IL-17A	Interleukin-17C	IL-15RA	Interleukin-15 receptor subunit alpha
IL-20	Interleukin-20	IL-17A	Interleukin-17A
IL-20RA	Interleukin-20 receptor subunit alpha	IL-17C	Interleukin-17C
IL-22RA1	Interleukin-22 receptor subunit alpha-1	IL-20	Interleukin-20
IL-24	Interleukin-24	IL-20RA1	Interleukin-20 receptor subunit alpha
IL-23	Interleukin-33	IL-22RA1	Interleukin-22 receptor subunit alpha-1
TSLP	Thymic stromal lymphopoietin	IL-24	Interleukin-24
SLAMF1	Signaling lymphocytic activation molecule family member 1	IL-33	Interleukin-33
TNF	Tumor necrosis factor	TSLP	Thymic stromal lymphopoietin
IFN-gamma	Interferon-gamma	TNF	Tumor necrosis factor
LIF	Leukemia inhibitory factor	IFN-gamma	Interferon-gamma
NRTN	Neurturin	LIF	Leukemia inhibitory factor
ST1A1	Sulfotransferase 1A1	NRTN	Neurturin
MCP-3	Monocyte chemotactic protein-3	ST1A1	Sulfotransferase 1A1
		MCP-3	Monocyte chemotactic protein-3
		PD-L1	Programmed cell death 1 ligand 1
		ARTN	Artemin
		GDNF	Glial cell line-derived neurotrophic factor

Supplementary Table S4. Seasonal variation of all inflammatory markers (n = 70) and the summary variable analyzed in the pregnancy sample, presented with both uncorrected and Bonferroni corrected *p*-value.

Inflammatory marker	Peak (day)		Trough (day)		Expected relative difference between trough and peak (%) [a]		Mean value		Uncorrected p-value		Bonferroni corrected p-value	
	ALL	NDS	ALL	NDS	ALL	NDS	ALL	NDS	ALL	NDS	ALL	NDS
101_IL_8	64	81	247	264	26.3	18.4	4.6	4.7	0.001	0.085	0.092	1.000
102_VEGF_A	79	84	262	267	20.5	22.1	10.1	10.2	0.000	0.000	<0.001*	<0.001*
106_hGDNF	79	87	262	270	21.5	19.3	1.6	1.6	0.000	0.008	0.032*	0.586
107_CDCP1	65	65	248	248	39.4	23.4	2.7	2.8	0.000	0.013	<0.001*	0.950
108_CD244	113	123	296	306	21.8	25.3	5.2	5.2	0.000	0.000	0.001*	0.002*
109_IL_7	61	89	244	272	25.2	28.3	2.1	2.1	0.009	0.008	0.630	0.561
110_OPG	77	84	260	267	33.0	28.4	10.5	10.6	0.000	0.001	<0.001*	0.099
111_LAP.TGF_beta_1	81	75	264	258	12.3	7.2	7.6	7.7	0.104	0.548	1.000	1.000
112_uPA	76	65	259	248	10.9	12.6	11.0	11.0	0.025	0.023	1.000	1.000
113_IL_6	60	72	243	255	37.5	36.3	2.3	2.3	0.001	0.005	0.072	0.345
114_IL_17C	56	43	239	226	19.1	32.7	1.7	1.7	0.025	0.004	1.000	0.300
115_MCP_1	86	99	269	282	20.0	18.2	9.0	9.0	0.000	0.003	0.003*	0.179
117_CXCL11	40	30	223	213	48.3	39.3	6.9	7.0	0.002	0.049	0.119	1.000
118_AXIN1	124	136	307	319	68.5	62.3	2.6	2.8	0.001	0.015	0.041*	1.000
120_TRAIL	77	96	260	279	13.0	12.8	7.3	7.3	0.006	0.018	0.410	1.000
122_CXCL9	37	26	220	209	44.5	43.2	5.1	5.2	0.002	0.036	0.174	1.000
123_CST5	82	100	265	283	17.0	18.7	5.1	5.2	0.001	0.003	0.089	0.189
126_OSM	102	137	285	320	26.7	35.6	3.7	3.7	0.067	0.058	1.000	1.000
128_CXCL1	42	45	225	228	35.8	28.4	7.8	7.8	0.008	0.105	0.588	1.000
130_CCL4	50	83	233	266	27.7	23.8	4.4	4.5	0.002	0.014	0.129	0.987
131_CD6	115	132	298	315	23.2	22.8	2.5	2.6	0.002	0.016	0.134	1.000
132_SCF	119	140	302	323	8.6	14.5	7.1	7.2	0.193	0.063	1.000	1.000
133_IL_18	82	78	265	261	23.7	28.1	7.5	7.5	0.004	0.008	0.274	0.537
135_TGFA	44	77	227	260	18.9	20.4	0.4	0.4	0.020	0.010	1.000	0.741
136_MCP_4	118	121	301	304	25.7	33.9	1.2	1.3	0.000	0.000	0.009*	0.002*
137_CCL11	52	44	235	227	17.8	14.1	6.0	6.1	0.021	0.219	1.000	1.000
138_TNFSF14	100	121	283	304	23.7	26.6	0.9	1.0	0.002	0.005	0.114	0.381
139_FGF_23	129	160	312	343	25.6	25.6	2.6	2.5	0.185	0.329	1.000	1.000
141_FGF_5	61	94	244	277	15.3	18.2	0.5	0.5	0.009	0.001	0.629	0.101

142_MMP_1	76	98	259	281	40.7	57.4	1.5	1.5	0.005	0.001	0.334	0.052
143_LIF_R	62	65	245	248	17.3	8.2	4.9	4.9	0.029	0.491	1.000	1.000
144_FGF_21	23	48	206	231	44.7	62.5	4.3	4.3	0.246	0.170	1.000	1.000
145_CCL19	66	65	249	248	38.3	46.2	8.2	8.3	0.001	0.001	0.050	0.044*
148_IL_15RA	92	113	275	296	16.7	17.2	0.2	0.3	0.000	0.000	0.005*	0.015*
149_IL_10RB	86	91	269	274	18.8	17.9	6.2	6.3	0.000	0.001	0.007*	0.085
151_IL_18R1	71	79	254	262	23.8	19.7	6.4	6.4	0.000	0.007	0.028*	0.493
152_PD_L1	64	91	247	274	20.3	26.1	3.3	3.3	0.017	0.008	1.000	0.562
153_Beta_NGF	91	100	274	283	18.6	20.5	0.8	0.9	0.000	0.000	0.001*	0.015*
154_CXCL5	72	96	255	279	23.1	30.1	9.1	9.1	0.366	0.319	1.000	1.000
155_TRANCE	119	116	302	299	25.1	34.8	2.1	2.1	0.001	0.000	0.060	0.012*
156_HGF	78	86	261	269	22.7	18.6	6.8	6.9	0.000	0.008	0.015*	0.566
157_IL_12B	90	99	273	282	26.4	25.4	3.1	3.1	0.000	0.009	0.021*	0.604
160_ARTN	83	78	266	261	22.7	24.7	0.8	0.9	0.000	0.001	0.022*	0.070
161_MMP_10	43	41	226	224	29.1	26.1	5.3	5.3	0.009	0.057	0.662	1.000
162_IL_10	38	46	221	229	23.9	17.8	2.5	2.6	0.023	0.262	1.000	1.000
164_CCL23	78	59	261	242	16.9	23.6	8.3	8.3	0.014	0.005	0.992	0.386
165_CD5	92	103	275	286	20.5	19.7	3.0	3.0	0.000	0.001	0.002*	0.067
166_MIP_1.alpha	81	104	264	287	25.6	29.7	1.8	1.8	0.001	0.003	0.087	0.209
167_Flt3L	66	66	249	249	10.3	11.9	8.5	8.5	0.089	0.113	1.000	1.000
168_CXCL6	84	94	267	277	34.6	30.0	6.3	6.4	0.001	0.021	0.081	1.000
169_CXCL10	41	44	224	227	43.0	36.3	8.6	8.6	0.002	0.044	0.145	1.000
170_4E_BP1	147	169	330	352	57.4	43.5	4.9	5.0	0.001	0.051	0.079	1.000
172_SIRT2	120	130	303	313	78.6	58.1	3.7	3.8	0.000	0.020	0.005*	1.000
173_CCL28	48	34	231	217	35.4	36.0	3.6	3.7	0.008	0.034	0.579	1.000
174_DNER	97	110	280	293	17.4	19.5	6.4	6.4	0.000	0.000	0.011*	0.025*
175_EN_RAGE	98	111	281	294	24.4	28.0	0.6	0.6	0.028	0.062	1.000	1.000
176_CD40	98	104	281	287	28.3	27.1	8.3	8.3	0.000	0.002	0.004*	0.127
179_FGF_19	40	60	223	243	30.4	27.7	7.1	7.2	0.056	0.132	1.000	1.000
183_MCP_2	61	60	244	243	45.7	53.0	7.5	7.5	0.000	0.000	0.001*	0.005*
184_CASP_8	118	128	301	311	27.2	24.5	0.6	0.7	0.003	0.060	0.222	1.000
185_CCL25	37	18	220	201	32.0	34.2	5.1	5.2	0.002	0.011	0.149	0.802
186_CX3CL1	55	45	238	228	20.2	17.9	5.0	5.1	0.001	0.021	0.068	1.000
187_TNFRSF9	63	57	246	240	24.4	24.1	5.3	5.4	0.000	0.000	<0.001*	0.026*
188_NT_3	74	58	257	241	32.2	32.6	1.6	1.7	0.001	0.011	0.102	0.805
189_TWEAK	98	126	281	309	12.5	14.9	7.5	7.6	0.009	0.013	0.625	0.908
190_CCL20	76	63	259	246	39.4	31.5	5.1	5.2	0.002	0.065	0.157	1.000

192_STAMBP	122	133	305	316	64.9	52.7	3.5	3.7	0.000	0.009	0.002*	0.630
194_ADA	109	104	292	287	17.5	10.4	4.7	4.8	0.030	0.420	1.000	1.000
195_TNFB	115	130	298	313	15.5	19.7	2.8	2.9	0.008	0.009	0.577	0.664
196_CSF_1	77	84	260	267	18.2	21.5	7.9	7.9	0.000	0.000	<0.001*	<0.001*
Summary variable	79	87	262	270	0.8	0.8	0.0	0.2	0.000	0.000	<0.001*	<0.001*

[a] Change in inflammation level (not NPX value, see methods for more details).

*Bonferroni corrected *p*-value < 0.05

NDS: no depressive symptoms

Supplementary Table S5. Seasonal variation of all inflammatory markers (n = 66) and the summary variable analyzed in the postpartum sample, presented with both uncorrected and Bonferroni corrected *p*-value.

Inflammatory marker	Peak (day)		Trough (day)		Expected relative difference between trough and peak (%) [a]		Mean value		Uncorrected p-value		Bonferroni corrected *p*-value	
	ALL	NDS	ALL	NDS	ALL	NDS	ALL	NDS	ALL	NDS	ALL	NDS
101_IL_8	165	186	348	3	15.5	20.4	4.8	4.7	0.314	0.327	1.000	1.000
102_VEGF_A	185	192	2	9	11.9	14.2	9.7	9.7	0.118	0.225	1.000	1.000
107_CDCP1	43	33	226	216	12.1	11.9	1.5	1.5	0.389	0.571	1.000	1.000
108_CD244	27	32	210	215	6.2	5.5	5.2	5.2	0.726	0.860	1.000	1.000
109_IL_7	190	213	7	30	11.0	19.1	1.7	1.7	0.559	0.409	1.000	1.000
110_OPG	39	74	222	257	10.8	7.9	9.3	9.3	0.322	0.459	1.000	1.000
111_LAP.TGF_beta_1	200	201	17	18	22.2	25.1	5.3	5.3	0.021	0.040	1.000	1.000
112_uPA	78	49	261	232	2.0	2.7	9.7	9.7	0.885	0.908	1.000	1.000
113_IL_6	291	227	108	44	8.8	30.3	1.5	1.4	0.681	0.319	1.000	1.000
115_MCP_1	190	195	7	12	13.0	19.6	9.1	9.0	0.101	0.042	1.000	1.000
117_CXCL11	177	186	360	3	41.6	31.4	6.5	6.4	0.029	0.197	1.000	1.000
118_AXIN1	154	176	337	359	45.8	66.0	2.2	2.3	0.093	0.091	1.000	1.000
120_TRAIL	239	197	56	14	7.7	4.7	7.5	7.5	0.290	0.713	1.000	1.000
122_CXCL9	40	27	223	210	31.2	56.0	5.6	5.6	0.281	0.095	1.000	1.000
123_CST5	148	168	331	351	6.2	8.3	4.9	4.9	0.612	0.578	1.000	1.000
126_OSM	250	248	67	65	58.2	61.0	2.0	2.0	0.007	0.027	0.483	1.000
128_CXCL1	171	106	354	289	30.2	11.0	7.6	7.6	0.120	0.726	1.000	1.000
130_CCL4	147	145	330	328	10.5	10.6	4.1	4.2	0.356	0.493	1.000	1.000
131_CD6	209	200	26	17	7.8	7.6	3.0	3.0	0.745	0.758	1.000	1.000
132_SCF	71	53	254	236	7.7	11.6	7.3	7.2	0.388	0.302	1.000	1.000
133_IL_18	225	230	42	47	13.1	7.9	6.7	6.6	0.533	0.825	1.000	1.000
134_SLAMF1	239	240	56	57	27.5	19.0	1.3	1.3	0.075	0.185	1.000	1.000
135_TGFA	233	221	50	38	13.5	22.9	0.0	0.0	0.140	0.067	1.000	1.000
136_MCP_4	194	204	11	21	37.3	44.7	1.8	1.8	0.001	0.002	0.045	0.110
137_CCL11	93	54	276	237	11.1	13.2	6.9	6.9	0.306	0.572	1.000	1.000

138_TNFSF14	199	201	16	18	29.1	37.1	0.6	0.6	0.027	0.025	1.000	1.000
139_FGF_23	36	13	219	196	1.3	11.8	1.6	1.5	0.992	0.571	1.000	1.000
141_FGF_5	362	335	179	152	4.8	4.8	0.5	0.5	0.769	0.805	1.000	1.000
142_MMP_1	138	124	321	307	33.2	21.4	0.9	1.0	0.034	0.349	1.000	1.000
143_LIF_R	43	52	226	235	10.9	11.2	1.8	1.8	0.215	0.261	1.000	1.000
144_FGF_21	23	362	206	179	35.6	9.2	2.7	2.6	0.487	0.937	1.000	1.000
145_CCL19	165	144	348	327	8.2	10.1	8.4	8.4	0.740	0.688	1.000	1.000
149_IL_10RB	249	245	66	62	15.3	21.0	5.0	5.0	0.067	0.016	1.000	1.000
151_IL_18R1	150	142	333	325	10.4	12.0	5.5	5.5	0.219	0.189	1.000	1.000
153_Beta_NGF	16	163	199	346	3.2	4.1	0.4	0.4	0.799	0.556	1.000	1.000
154_CXCL5	110	82	293	265	38.7	15.6	9.8	9.9	0.169	0.821	1.000	1.000
155_TRANCE	173	180	356	363	8.9	11.1	3.3	3.3	0.604	0.656	1.000	1.000
156_HGF	260	269	77	86	10.2	9.9	5.5	5.5	0.227	0.320	1.000	1.000
157_IL_12B	212	203	29	20	14.7	14.5	3.6	3.6	0.467	0.582	1.000	1.000
161_MMP_10	126	60	309	243	6.4	6.0	5.4	5.3	0.829	0.916	1.000	1.000
162_IL_10	315	318	132	135	20.2	19.6	2.2	2.2	0.052	0.161	1.000	1.000
164_CCL23	324	39	141	222	5.4	2.4	9.1	9.1	0.628	0.978	1.000	1.000
165_CD5	229	239	46	56	19.9	15.4	3.2	3.2	0.030	0.092	1.000	1.000
166_MIP_1.alpha	179	190	362	7	10.0	14.9	1.4	1.4	0.324	0.262	1.000	1.000
167_Flt3L	196	206	13	23	7.4	12.5	8.1	8.1	0.626	0.460	1.000	1.000
168_CXCL6	188	197	5	14	41.2	31.3	6.4	6.4	0.027	0.251	1.000	1.000
169_CXCL10	320	10	137	193	7.9	9.8	8.1	8.1	0.814	0.860	1.000	1.000
170_4E_BP1	195	207	12	24	49.7	58.8	4.5	4.5	0.039	0.067	1.000	1.000
172_SIRT2	182	185	365	2	72.2	71.7	2.9	2.9	0.019	0.061	1.000	1.000
173_CCL28	122	12	305	195	3.0	4.4	1.5	1.6	0.901	0.926	1.000	1.000
174_DNER	37	35	220	218	3.3	6.1	6.2	6.2	0.788	0.561	1.000	1.000
175_EN_RAGE	224	220	41	37	36.3	42.6	0.8	0.8	0.045	0.052	1.000	1.000
176_CD40	150	149	333	332	12.0	14.9	7.9	7.9	0.178	0.141	1.000	1.000
179_FGF_19	213	195	30	12	34.3	41.8	7.1	7.1	0.286	0.231	1.000	1.000
183_MCP_2	185	172	2	355	26.3	27.8	7.8	7.8	0.178	0.207	1.000	1.000

184_CASP_8	177	167	360	350	33.3	28.3	0.2	0.2	0.008	0.007	0.508	0.464
185_CCL25	64	45	247	228	21.7	26.8	5.2	5.2	0.184	0.227	1.000	1.000
186_CX3CL1	230	228	47	45	7.1	10.3	4.9	4.8	0.646	0.511	1.000	1.000
187_TNFRSF9	188	171	5	354	6.5	6.0	5.4	5.4	0.556	0.603	1.000	1.000
188_NT_3	154	120	337	303	26.2	36.4	1.8	1.7	0.005	0.000	0.304	0.033*
189_TWEAK	170	210	353	27	2.7	3.8	8.4	8.4	0.837	0.865	1.000	1.000
190_CCL20	68	37	251	220	32.0	28.6	5.5	5.5	0.169	0.483	1.000	1.000
192_STAMBP	169	178	352	361	57.2	60.1	3.0	3.0	0.007	0.022	0.453	1.000
194_ADA	167	110	350	293	17.7	10.3	4.4	4.4	0.083	0.163	1.000	1.000
195_TNFB	236	32	53	215	1.2	1.0	2.8	2.7	0.990	0.995	1.000	1.000
196_CSF_1	199	202	16	19	5.7	8.1	6.9	6.9	0.433	0.356	1.000	1.000
Summary variable	163	164	346	347	0.4	0.3	-0.0	-0.0	0.182	0.313	1.000	1.000

[a] Change in inflammation level (not NPX value, see methods for more details).

* Bonferroni corrected *p*-value < 0.05

NDS: no depressive symptoms
